# Arctigenin Attenuates Hepatic Stellate Cell Activation via Endoplasmic Reticulum-Associated Degradation (ERAD)-Mediated Restoration of Lipid Homeostasis

**DOI:** 10.1021/acs.jafc.5c01366

**Published:** 2025-05-26

**Authors:** Mengmeng Xia, Jia Li, Lizbeth Magnolia Martinez Aguilar, Junyu Wang, Maria Camila Trillos Almanza, Yakun Li, Manon Buist-Homan, Han Moshage

**Affiliations:** Department of Gastroenterology and Hepatology, University Medical Center Groningen, University of Groningen, Groningen 9713 GZ, The Netherlands

**Keywords:** arctigenin, hepatic stellate cell activation, endoplasmic reticulum stress, lipid droplet homeostasis, endoplasmic reticulum-associated degradation

## Abstract

Arctigenin, a natural lignan from Arctium lappa L., exhibits potent antifibrotic activity, yet its molecular mechanisms remain unclear. Endoplasmic reticulum (ER) stress is known to promote hepatic stellate cell (HSC) activation and liver fibrosis. This study investigates the therapeutic potential of arctigenin in HSC activation through ER stress modulation. Primary rat HSCs were activated (3–7 days) and treated with tunicamycin (ER stress inducer) or 4-PBA (ER stress inhibitor). Arctigenin attenuated ER stress markers (e.g., GRP78) and suppressed the expression of fibrotic marker α-SMA in ER stress-challenged activating (day 3) and activated (day 7) HSCs. Arctigenin restored lipid homeostasis by modulation of both lipogenesis (*via* Dgat2 and Ppar-γ upregulation) and lipolysis (suppression *via* ATGL inhibition). ER stress activated ER-associated degradation (ERAD), triggering the formation of small lipid droplets (LD). Arctigenin normalized the ERAD activity, thereby rescuing LD integrity and suppressing HSC activation. Our findings demonstrate that arctigenin mitigates HSC activation by suppressing ER stress and restoring lipid homeostasis *via* modulating ERAD-mediated lipid dysregulation. As a dietary and medicinal compound, arctigenin emerges as a promising therapeutic candidate for liver fibrosis.

## Graphical abstract

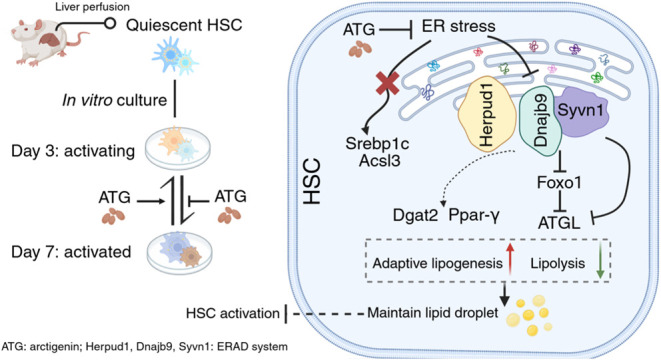

## 1 Introduction

Liver fibrosis is a dynamic pathological process resulting from chronic liver disease, including metabolic dysfunction-associated steatohepatitis, alcohol-related liver disease, and hepatitis C or B viral infections.^1^ Advanced liver fibrosis progresses to cirrhosis, hepatocellular carcinoma, and ultimately liver failure, the leading cause of liver transplantation.^2^ The onset and development of liver fibrosis is characterized by the accumulation of excessive extracellular matrix proteins (ECM) in the liver, particularly the deposition of collagen types I and III.^3^ Activated hepatic stellate cells (aHSCs) synthesize excessive amounts of the extracellular matrix and contribute to fibrotic scar formation in response to chemokine and cytokine stimulation from inflammatory or injured cells. Therefore, aHSCs are recognized as the primary drivers of liver fibrosis, and inhibition and reversal of HSC activation have been proposed as novel therapeutic strategies for liver fibrosis.^4,5^

The endoplasmic reticulum (ER) is an important compartment for protein biosynthesis, folding, and trafficking in cells, and many proteins are involved in regulating proteostasis and ER homeostasis.^6^ When the protein-folding demand exceeds ER capacity or when ER function is compromised, the accumulation of misfolded proteins triggers ER stress.^6,7^ The Unfolded Protein Response (UPR) is the initial response to ER stress and serves to restore ER homeostasis by increasing the production of proteins related to the protein-folding capacity of the ER.^8^ When the UPR is unable to restore protein-folding capacity, persistent ER stress triggers cellular damage and apoptotic cell death in various cell types, initiating pathogenic cascades implicated in multiple human diseases.^9,10^ Accumulating evidence has demonstrated that ER stress as an inducer of HSCs activation drives hepatic fibrogenesis.^8,11,12^ Chronic ER stress induces paradoxical cellular responses in HSCs: ER stress induces apoptosis^12^ but also promotes HSC transdifferentiation into fibrogenic myofibroblast-like cells *via* the IRE1α-dependent p38/MAPK and PERK-associated UPR signaling pathways.^8,13,14^ This ER stress-mediated phenotype facilitates excessive ECM deposition and creates a pro-fibrotic microenvironment that perpetuates hepatic scarring.^15^ Clinical analysis reveals coordinated upregulation of UPR markers, e.g., Grp78 and Chop, and HSC activation markers, e.g., α-SMA, in fibrotic human livers, suggesting the spatial correlation between ER stress hotspots and fibrotic regions.^8^ Importantly, pharmacological induction of ER stress accelerates HSC activation in both *in vitro* models and *in vivo* murine models.^8,16^ These studies indicate that the modulation of ER stress is a valid therapeutic strategy against liver fibrosis.

ER-associated degradation (ERAD) is a conserved pathway that eliminates misfolded ER proteins *via* the ubiquitin-proteasome system, preserving ER homeostasis.^6^ ERAD operates by recognizing misfolded substrates, translocating them across the ER lipid bilayer into the cytoplasm, and facilitating their degradation *via* membrane-associated E2/E3 ubiquitin ligases such as SYVN1 and GP78.^17^ Emerging evidence implicates ERAD in the pathogenesis of liver fibrosis, where the E3 ligase SYVN1 has been identified as essential for HSC activation.^18^ SYVN1 suppression attenuates the progression of fibrosis,^18^ indicating that ERAD is a pivotal regulator of this process. ERAD also plays a central role in lipid droplet (LD) homeostasis, since the ER mediates LD biogenesis.^19^ LDs are ubiquitous organelles that store lipids for energy production or membrane synthesis. It also acts as a hub for various metabolic processes.^20,21^ ERAD functions as a rate-limiting factor in LD formation, since it regulates conversion of diacylglycerol (DAG) to triacylglycerol (TAG) through the ERAD sensor UbxD8 and its membrane partner Ubx2, thereby fine-tuning neutral LD biosynthesis.^22,23^ Inhibition of ERAD ubiquitin ligases increases TAG synthesis enzymes GPAT3, MOGAT2, and DGAT2, resulting in increased fatty acid re-esterification and tissue triacylglycerol synthesis, leading to increased LD formation and accumulation.^24^ In addition, the ERAD adaptor facilitates lipase maturation and secretion, promoting LD mobilization.^25^ As a major site of lipid storage in the liver, quiescent (non-activated) HSCs store mainly retinoids and neutral LDs.^26^ Upon activation, the quiescent HSCs lose their LDs as a marker of ER stress increase,^8,26^ suggesting that the interplay among ER stress, ERAD, and LD formation are significant in HSC activation. Consequently, modulation of this interplay may restore LD content in HSCs and reverse their pro-fibrotic activation.

Arctigenin is a bioactive lignan extracted from Fructus arctii, the dried, ripe fruit of Arctium lappa L.,^27^ which is a medicine and food homologue (MFH) present in functional foods due to its dual dietary and therapeutic potential. Emerging evidence highlights the multifaceted pharmacological activities of arctigenin, particularly in ameliorating ER stress through AMPK activation and suppression of the unfolded protein response (UPR).^28–30^ Despite these findings, the therapeutic properties of arctigenin on ER stress-driven liver fibrogenesis remain unexplored, with systematic investigations lacking in both *in vitro* and *in vivo* models. In the present study, we used an *in vitro* HSC activation model to investigate the antifibrotic mechanisms of arctigenin with a focus on ER stress modulation and its interplay with the ERAD system. Our findings reveal that arctigenin suppresses HSC activation by restoring ERAD-mediated lipid homeostasis. This discovery not only elucidates a novel molecular mechanism underlying the antifibrotic activity of arctigenin but also establishes a theoretical foundation for its dual applicability as both a functional food ingredient and a therapeutic agent for liver fibrosis.

## 2 Materials and Methods

### 2.1 Primary Rat Hepatic Stellate Cell Isolation and Culture

Adult male Wistar rats (250–300 g) were purchased from Charles River Laboratories Inc. (Wilmington, MA) and housed in the central animal facility of the University Medical Center Groningen. All animals had *ad libitum* access to food and water. The animal procedures in this study were approved by the Animal Welfare Body of the University of Groningen under an ethical license No. 2115139-01-001. Primary hepatic stellate cells (HSCs) were isolated from rats as previously described.^31^ Briefly, livers were perfused *in situ* with Pronase (Merck, Amsterdam, The Netherlands) followed by collagenase P (Roche, Almere, The Netherlands). Dispersed cell suspensions were layered on a 13% Nycodenz density gradient (Axis-Shield POC, Oslo, Norway), and HSCs were subsequently separated by density gradient centrifugation. Freshly isolated HSCs were cultured in Iscove’s Modified Dulbecco’s Medium (IMDM; Thermo Fisher Scientific, Breda, The Netherlands) supplemented with 20% fetal bovine serum, 1% MEM nonessential amino acids, 1% sodium pyruvate (all from Thermo Fisher Scientific), and antibiotics: 50 μg/mL gentamycin (Thermo Fisher Scientific), 100 units/mL streptomycin (Lonza, Vervier, Belgium), 100 units/mL penicillin (Lonza), and 250 ng/Ml Fungizone (Lonza). HSCs were maintained at 37 °C in a humidified atmosphere containing 5% CO_2_. HSCs isolated on day 1 exhibit a quiescent phenotype characterized by abundant retinoid-containing lipid droplets (LDs; Figure S4a). Upon culture on plastic surfaces, spontaneous progressive activation occurs: by day 3, HSCs transitioned to an activating stage,^32^ i.e., intermediate activation state, retaining partial LDs but displaying reduced retinoid storage (Figure S4a). By day 7, cells reached full activation,^32^ marked by complete LD depletion and concurrent declines in neutral lipids (e.g., triglycerides).^26^ This activation cascade correlated with elevated expression of α-smooth muscle actin (α-SMA, indicative of myofibroblastic transdifferentiation) and collagen type I α1 (Col1α1), a hallmark of extracellular matrix synthesis.^26^ Additionally, progressive increased proliferation is observed during this activation process.

### 2.2 Chemicals

Arctigenin (purity >98%) was obtained from Dilger Medicine (Nanjing, China), Tunicamycin from *Streptomyces sp*. and sodium 4-phenylbutyrate (4-PBA) were from Sigma-Aldrich (Zwijndrecht, The Netherlands), and Eeyarestatin-I (Eer I) was purchased from Santa Cruz (Heidelberg, Germany).

### 2.3 Experimental Design

This study investigated the antifibrotic effects of arctigenin on HSC activation, focusing on its protective effect against ER stress-driven lipid dysregulation. Freshly isolated primary HSCs were seeded at 1 × 10^6^ cells/mL and cultured for 3 days (activating phase) or 7 days (fully activated state), as outlined in Figure S1. In phase 1, activating and activated HSCs were treated with ATG to evaluate the dose-dependent suppression of proliferation, activation markers (α-SMA, Col1a1), and ER stress mediators (Grp78). Phase 2 employed ER stress modulation, activating HSCs (day 3) were exposed to tunicamycin (2 μg/mL, 12 h) to induce ER stress, while activated HSCs (day 7) received 4-PBA (3 mM, 12 h) to alleviate ER stress. The ability of arctigenin to ameliorate ER stress-driven HSC activation was validated by assessing the lipid droplet dynamics and the analysis of lipid metabolism pathways, confirming its role in restoring lipid homeostasis. Phase 3 mechanistically linked ERAD to lipid regulation by inhibiting ERAD with Eeyarestatin-I (Eer I), demonstrating that arctigenin attenuates HSC activation through the modulation of ERAD activity.

### 2.4 Cell Toxicity Assay

Necrotic cells were determined using SYTOX Green nucleic acid staining (Thermo Fisher Scientific) at a dilution of 1:40,000 in Hanks’ Balanced Salt Solution for 15 min at 37 °C under CO_2_-free conditions. Hydrogen peroxide (1 mmol/L) was used as a positive control for necrosis. Fluorescent nuclei were visualized using a Leica microscope (Amsterdam, The Netherlands) equipped with an excitation filter 450–490 nm bandpass (Leica #11513867, Chroma ET470/40x equivalent).

Quantification of cytotoxicity was evaluated by a WST-1 assay (Roche). About 30,000 HSCs were seeded per well in 96-well plates and treated with arctigenin for 48 h. 10 μL of WST-1 solution per 100 μL of culture medium was added to each well and incubated for 2 h at 37 °C. The absorbance was quantified using a microreader (Bio-Tek, Winooski, VT).

### 2.5 Cell Proliferation Measurement

HSC proliferation was measured using a Real-Time xCELLigence system (RTCA DP; ACEA Biosciences Inc., Santa Clara, CA). Around 3000–5000 HSCs were seeded per well in a 16-well E-plate (Agilent, Santa Clara, CA) and treated with arctigenin. Normalized cell index was determined by measuring real-time cellular impedance.

### 2.6 Detection of Intracellular Neutral Lipid Droplets

HSCs were seeded on coverslips in 12-well plates and treated as described. Neutral lipid droplets (LDs) inside HSCs were visualized using BODIPY-LD staining as previously reported.^33^ Briefly, cells were fixed with 3.7% paraformaldehyde solution (Merck Darmstadt, Germany) for 15 min, followed by washes with phosphate-buffered saline (PBS, Thermo Fisher Scientific). LDs were fluorescently labeled with BODIPY 493/503 dye (1:2500 dilution in PBS, Thermo Fisher Scientific) for 15 min. Nuclei were subsequently counterstained with 4′,6-diamidino-2-phenylindole (DAPI, Sigma-Aldrich) for 10 min. Cell imaging was performed using a fluorescent microscope (Leica, Amsterdam, The Netherlands).

### 2.7 Detection of Vitamin A (Retinoids) Autofluorescence

HSCs were cultured in 12-well plates, and intracellular vitamin A autofluorescence was determined using a fluorescence microscope (Leica) with an excitation filter of 365 nm.

### 2.8 Live-Cell Imaging of Endoplasmic Reticulum (ER)

HSCs were cultured on coverslips and treated with the ER stress inducer tunicamycin and the ER stress-relieving compounds 4-PBA and arctigenin. After the treatment, HSCs were washed 3 times with PBS (Thermo Fisher Scientific) to remove cell debris and then incubated with 1 μmol/L ER-tracker (Thermo Fisher Scientific) for 30 min in the dark. After incubation, HSCs were stained with Hoechst (Thermo Fisher Scientific) for 10 min. Fluorescent ER was detected using a microscope (Leica) at an excitation wavelength of 587 nm.

### 2.9 Quantitative Real-Time Reverse Transcription Polymerase Chain Reaction

HSC RNA was extracted using TriReagent (Sigma-Aldrich) according to the manufacturer’s protocol. RNA quality and quantity were measured by using a Nano-Drop 2000c spectrophotometer (Thermo Fisher Scientific). 1.5 μg portion of RNA was used for reverse transcription using M-MLV polymerase (Thermo Fisher Scientific). Quantitative real-time polymerase chain reaction was conducted using the QuantStudio 3 system (Thermo Fisher Scientific). The mRNA levels of Plin2, Pnpla2, Cyp26a1, Syvn1, Dnajc10, Dnajb9, and Herpud1 were quantified using commercial tests (Thermo Fisher Scientific), and other genes were quantified using the Taqman primers and probes listed in Table S1. All samples were analyzed in duplicate. Relative mRNA expression was calculated using the 2^–ΔΔCt^ method, with 36b4 as the normalizing gene.

### 2.10 Western Blot Analysis

After treatment, HSCs were collected using lysis buffer (HEPES 25 mmol/L, KAc 150 mmol/L, EDTA 2 mmol/L, NP-40 0.1%, NaF 10 nmol/L, PMSF 50 mmol/L, aprotinin 1 μg/μL, pepstatin 1 μg/μL, leupeptin 1 μg/μL, and DTT 1 mmol/L). Total protein in HSCs was quantified using the Bio-Rad protein assay (Bio-Rad, Hercules, CA). About 20–30 μg of protein was separated by SDS-PAGE gels and transferred to nitrocellulose membranes (Amersham, Piscataway, NJ) using a Trans-Blot Turbo Blotting system (Bio-Rad). Protein was detected using the following primary antibodies: anti-Actin α-Smooth Muscle (A5228, Sigma-Aldrich, 1:2000), anti-COL1A1 (1310-01, Southern Biotech, 1:1000, Birmingham, AL), anti-BiP (also called anti-Grp78, 3183, Cell Signaling Technology, 1:1000, Leiden, The Netherlands), anti-P21 (ab109199, Abcam, 1:1000), anti-phospho-HSL (4126, Cell Signaling Technology, 1:1000), anti-HSL (4107, Cell Signaling Technology, 1:1000), anti-ATGL (2138, Cell Signaling Technology, 1:1000), anti-phospho-eIF2α (9721, Cell Signaling Technology, 1:1000), anti-eIF2α (9722, Cell Signaling Technology, 1:1000), and anti-Tubulin (T9026, Sigma-Aldrich, 1:1000). Protein signals were detected by using a ChemiDoc MP Imaging system (Bio-Rad).

### 2.11 Statistical Analysis

All data are presented as means ± standard deviation (means ± SD). Every experiment was repeated at least three times using HSCs from different isolations. Statistical analysis was performed with GraphPad Prism (V..8.0.1, GraphPad Software, San Diego, CA). One-way analysis of variance (ANOVA) test followed by Tukey’s multiple comparison tests was used to evaluate group differences. *P* values < 0.05 were considered statistically significant.

## 3 Results and Discussion

### 3.1 Arctigenin Inhibits Hepatic Stellate Cell Activation *In Vitro*

Primary rat hepatic stellate cells (rHSCs) were activated *via* a standard plastic adherence culture, a validated experimental model for recapitulating spontaneous *in vitro* activation. To assess the toxicity of arctigenin, cells at distinct activation stages, early activating (day 3) and fully activated myofibroblasts (day 7, denoted as aHSCs in figures), were exposed to arctigenin (1–50 μM) for 48 h (experimental design detailed in Figure S1a). This concentration range was selected based on prior evidence of its bioactivity in HSCs,^34^ spanning concentrations with demonstrated biological activity.

Membrane integrity, a definitive criterion for distinguishing viable and nonviable cells *in vitro*, was evaluated using SYTOX Green. This fluorescent dye selectively binds nucleic acids upon loss of membrane integrity.^35^ Hydrogen peroxide (H_2_O_2_) was used as a positive control for the loss of membrane permeability (Figure S2a,b). Arctigenin treatment (1–50 μM) induced no detectable SYTOX Green uptake in HSCs (Figure S2a,b). Cellular viability, reflecting metabolic competence, was also quantified *via* the WST-1 assay.^36^ As shown in Figure S2c,d, the viability of activating and activated HSCs treated with arctigenin (1–50 μM) remained comparable to that of untreated controls (∼100%), confirming the absence of cytotoxicity across all tested concentrations. These data collectively demonstrate that arctigenin exhibits no cytotoxic effects on HSCs, irrespective of their activation status (activating or fully activated).

Under normal conditions, HSCs remain in a quiescent, non-proliferative state; however, upon activation, they become highly proliferative.^3^ This expansion of the activated HSC population drives ECM deposition, thereby accelerating fibrosis progression.^37^ Consequently, the pharmacological inhibition of HSC proliferation emerges as a promising therapeutic strategy for hepatic fibrosis management. In the present study, we employed the xCELLigence real-time cell analysis system to quantitatively assess proliferative dynamics through continuous monitoring of impedance variations in specialized microelectronic plates^38^ (experimental design illustrated in Figure S1b). This label-free methodology enables precise measurement of cellular spreading and the number of HSCs, offering distinct advantages over conventional end point quantification approaches. Our experimental design captured proliferation profiles during critical transitional phases: early activating and fully activated states (Figure S1b), with data acquisition completed within a 48 h observation window. Notably, administration of arctigenin significantly attenuated the real-time cell index in early activating HSCs compared to the control group (Figure 1a). This inhibitory effect persisted in fully activated HSCs, where increasing arctigenin concentrations produced a progressive reduction in proliferative indices (Figure 1b). These findings collectively demonstrate the efficacy of arctigenin in inhibiting HSC proliferation regardless of their activation state, suggesting its potential for therapeutic intervention at multiple stages of fibrotic progression.

**Figure 1:**
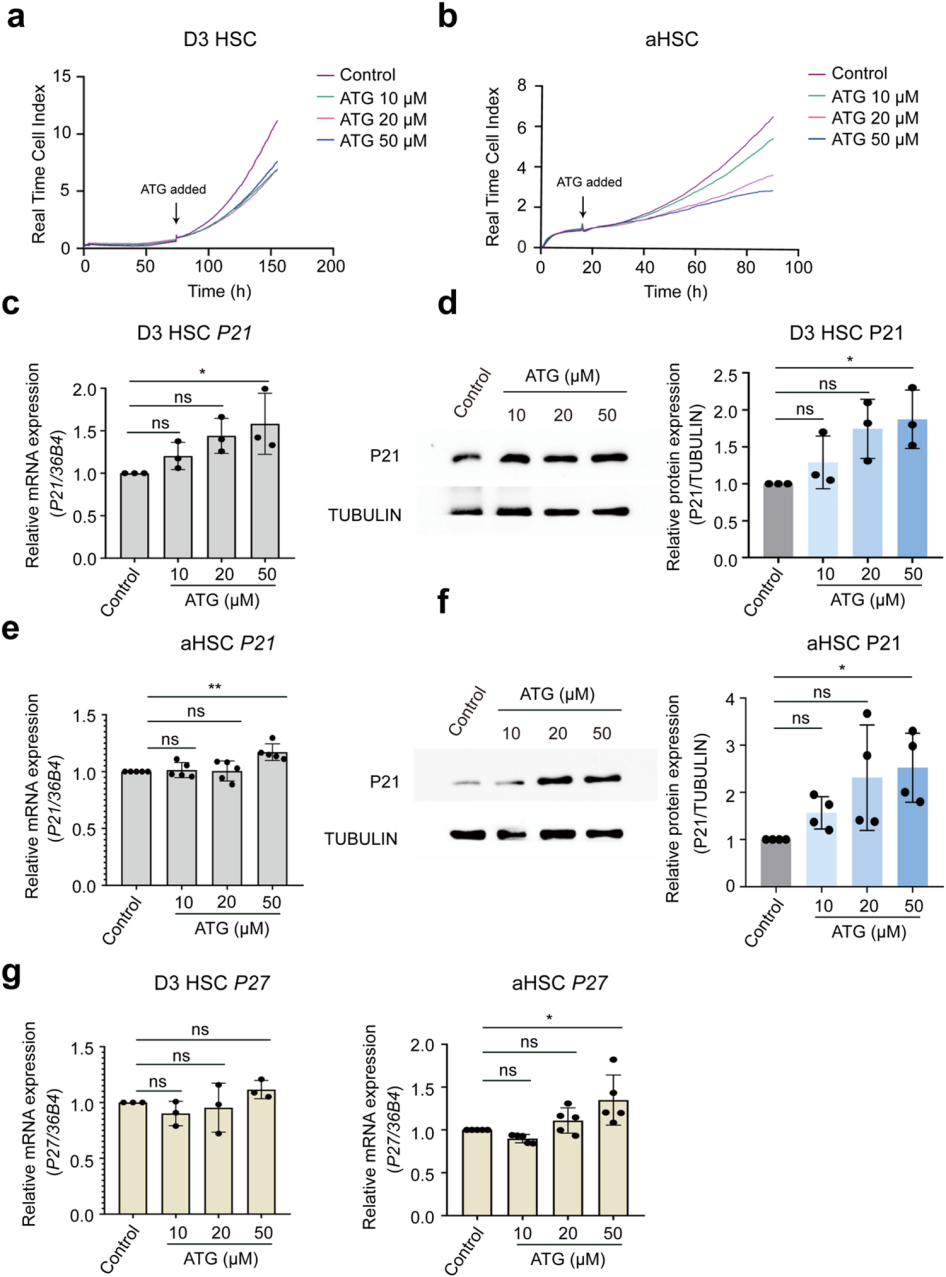
Arctigenin inhibits hepatic stellate cell proliferation *in vitro*. Activating (Day 3) hepatic stellate cells (HSCs) and activated (Day 7) HSCs (aHSCs) were treated with arctigenin (10, 20, and 50 μM) for 48 h. (a, b) Proliferation of activating and activated HSCs was quantified using the xCELLigence real-time cell index system. (c) Quantitative real-time PCR (qPCR) analysis of mRNA expression of the cell cycle inhibitor p21^Cip1^ in activating HSCs following 48 h arctigenin treatment. (d) Western blot analysis of p21^Cip1^ protein expression in activating HSCs treated with arctigenin for 48 h. Protein band intensities were quantified using ImageJ software. (e) qPCR analysis of p21^Cip1^ mRNA expression in activated HSCs after 48 h of arctigenin treatment. (f) Western blot analysis of p21^Cip1^ protein expression in activated HSCs treated with arctigenin for 48 h. Protein band intensities were quantified using ImageJ software. (g) qPCR analysis of p27^Kip1^ mRNA expression in activating and activated HSCs following 48 h arctigenin treatment. HSC, Hepatic stellate cell; D3 HSC, activating HSCs; aHSC, D7 (fully) activated HSCs; ATG, arctigenin. Data are presented as mean ± standard deviation (mean ± SD, *n* ≥ 3 per group). Statistical significance was determined by one-way ANOVA followed by Tukey’s post hoc test. Significance: ns: not significant, *P* > 0.05; *: *P* < 0.05; **: *P* < 0.01.

Pharmacological suppression of cellular proliferation may result from compromised cellular functionality.^39^ Notably, the absence of cytotoxicity observed in arctigenin-treated HSCs demonstrates that its antiproliferative effects are not mediated through cellular dysfunction or cytotoxic damage (Figure S2). A critical regulatory mechanism governing cell cycle progression involves the activation of cyclin-dependent kinase inhibitors (CKIs), which modulate cyclin protein-CDK complexes, thereby leading to cell cycle arrest.^40^ Contrary to LX2 cells, where arctigenin induced P27 ^Kip1^-mediated cell cycle arrest,^41^ our study revealed that 50 μM arctigenin upregulated both mRNA and protein levels of P21^Cip1^ in activating and fully activated HSC populations, whereas lower concentrations failed to elicit this response (Figure 1c–f). Interestingly, P27^Kip1^ expression remained unaltered by 50 μM arctigenin in activating HSCs but exhibited a significant elevation in activated HSCs (Figure 1g). The divergence between our results and prior reports could be attributed to phenotypic heterogeneity in HSC activation states and/or interspecies variations between rodent and human cell models, and the fact that LX-2 is an immortalized cell line.^42^ This study further underscores the dynamic regulation of cell cycle progression during HSC activation, wherein distinct CDK inhibitors may govern the proliferative capacity at different differentiation stages. Such stage-specific regulatory mechanisms warrant consideration when developing targeted antifibrotic therapies.

Quiescent HSCs undergo a dramatic phenotypic transition into myofibroblasts in response to hepatic injury. This phenotypic alteration is identified by an elevated expression of α-smooth muscle actin (α-SMA), a key biomarker associated with HSC activation, fibrogenic progression, and fibrosis severity in MASLD patients. Our findings demonstrate that 50 μM arctigenin significantly attenuated both mRNA and protein expression of α-SMA in activating and activated HSCs compared to their corresponding controls (Figure 2a–d), whereas lower concentrations (10–20 μM) exhibited no significant effects (Figure 2a–d). The antifibrotic efficacy of arctigenin was corroborated by parallel reductions in collagen type I α 1 (Col1a1) expression, a predominant ECM component overproduced by activated HSCs during fibrogenesis. In activating HSCs, arctigenin induced dose-dependent suppression of Col1a1 mRNA and protein levels across the 10–50 μM concentration range (Figure 2a,b). In fully activated HSCs, significant Col1a1 inhibition required higher therapeutic thresholds, with both 20 and 50 μM concentrations achieving inhibitory efficacy (Figure 2c,d). The antifibrotic potential of arctigenin was further substantiated through immunofluorescence analyses, revealing marked attenuation of α-SMA abundance specifically at 50 μM arctigenin in activating and activated HSCs and progressive Col1a1 reduction with increasing arctigenin concentrations (Figure 2e,f). Collectively, these observations demonstrate that arctigenin treatment displays antifibrotic effects by blocking HSC proliferation and suppressing HSC activation markers (α-SMA, Col1a1). The antiproliferative effects of arctigenin during HSC activation may operate through mechanisms dependent on canonical Cip/Kip-mediated cell cycle arrest, given the upregulation of specific CKIs.

**Figure 2:**
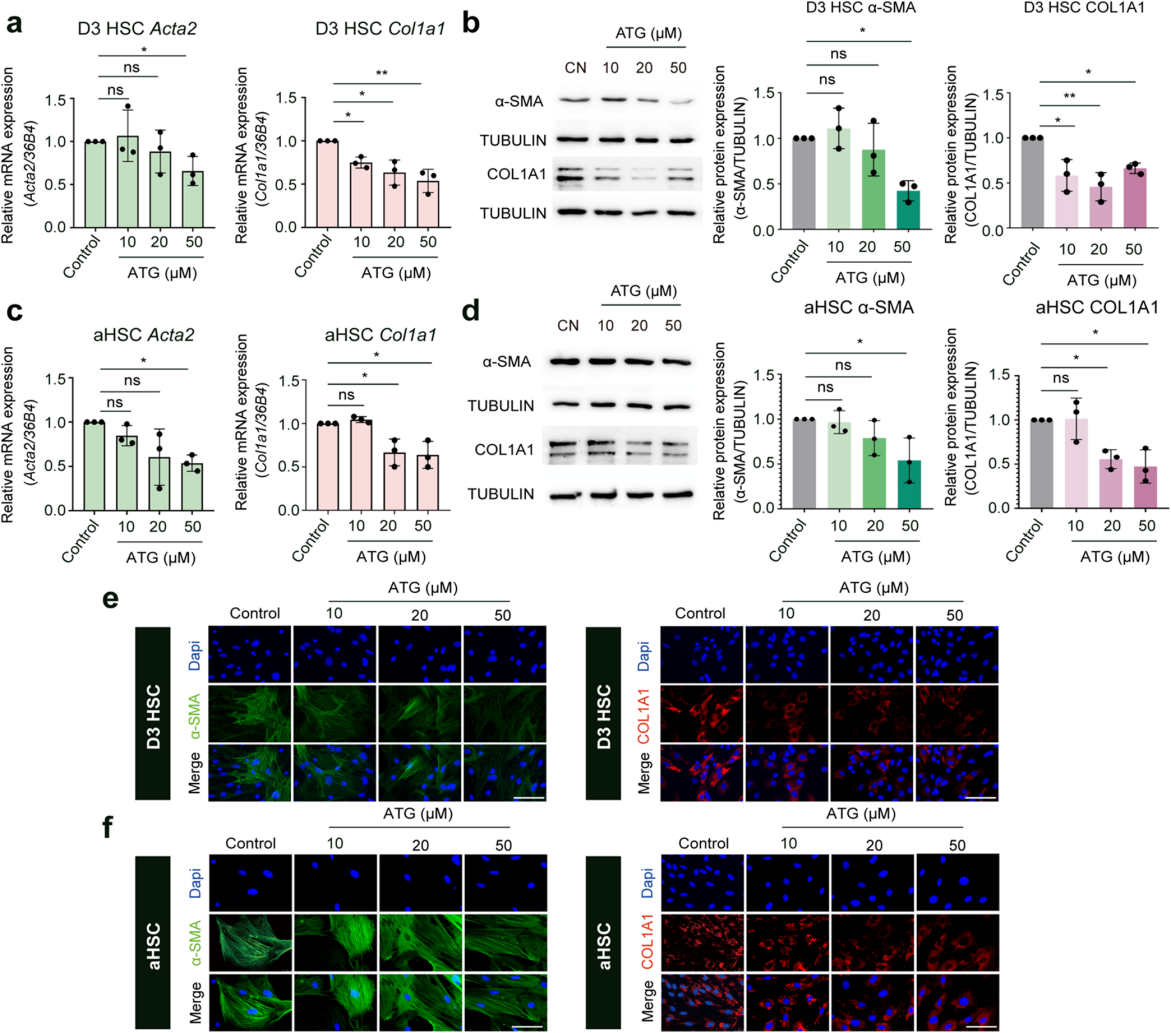
Arctigenin inhibits hepatic stellate cell activation *in vitro*. Activating (Day 3) HSCs and activated (Day 7) HSCs (aHSCs) were treated with arctigenin (10, 20, and 50 μM) for 48 h. (a) Quantitative real-time PCR (qPCR) analysis of mRNA expression for the HSC activation marker α-smooth muscle actin (α-SMA) and extracellular matrix (ECM) molecule collagen type I α 1 (Col1α1) in activating HSCs treated with arctigenin for 48 h. (b) Western blot analysis of α-SMA and Col1α1 protein expression in activating HSCs treated with arctigenin for 48 h. Protein band intensities were quantified using ImageJ software. (c) qPCR analysis of α-SMA and Col1α1 mRNA expression in avtivated HSCs treated with arctigenin for 48 h. (d) Western blot analysis of α-SMA and Col1α1 protein expression in activated HSCs treated with arctigenin for 48 h. Protein band intensities were quantified using ImageJ software. (e) Immunofluorescence analysis of α-SMA and Col1α1 expression in activating HSCs treated with arctigenin for 48 h. Scale bar, 100 μm. (f) Immunofluorescence analysis of α-SMA and Col1α1 expression in activated HSCs treated with arctigenin for 48 h. Scale bar: 100 μm. HSC, Hepatic stellate cell; D3 HSC, activating HSCs; aHSC, D7 (fully) activated HSCs; ATG, arctigenin. Data are presented as mean ± standard deviation (mean ± SD, *n* = 3 per group). Statistical significance was determined by one-way ANOVA followed by Tukey’s post hoc test. Significance: ns: not significant, *P* > 0.05; *: *P* < 0.05; **: *P* < 0.01.

### 3.2 Arctigenin Suppresses ER Stress-Induced Hepatic Stellate Cell Activation

ER stress is known to promote HSC activation and increase the expression of UPR markers in fibrotic liver.^8,12^ Therefore, alleviation of ER stress using functional food-derived bioactive molecules, particularly those modulating the UPR, may constitute an innovative therapeutic strategy for hepatic fibrosis intervention. To investigate the therapeutic efficacy of arctigenin against ER stress during HSC activation, we first quantified Grp78 levels, which are canonical UPR biomarkers across progressive HSC activation phases. As illustrated in Figure 3a,b, arctigenin treatment at 20 and 50 μM concentrations significantly reduced Grp78 mRNA and protein expression in activating HSCs, while 50 μM arctigenin effectively suppressed Grp78 in fully activated HSCs. Live-cell imaging using ER-Tracker dye revealed dose-dependent attenuation of ER fluorescence intensity in both activation stages following arctigenin intervention (Figure 3c,d), suggesting that arctigenin mediates the suppression of ER expansion. We further verified this effect in pharmacological ER stress-exposed HSC models: Day 3 HSCs (activating phase) were challenged with tunicamycin to exacerbate ER stress. Day 7 HSCs (completely activated) received 4-PBA to ameliorate ER stress (Figure S1c,d). The UPR involves three ER membrane sensors: IRE1 (inositol-requiring transmembrane kinase/endoribonuclease), PERK (double-stranded RNA-dependent protein kinase-like eukaryotic initiation factor 2a [eLF2a] kinase), and ATF6 (activating transcription factor 6). Under physiological conditions, these sensors remain inactive through binding to the ER chaperone Grp78.^7^ ER stress triggers their activation through Grp78 dissociation, initiating the UPR cascade signaling. IRE1 leads to the activation of downstream Xbp1s and Chop, PERK facilitates the expression of Atf4 and phosphorylation of eIF2α, whereas dissociated ATF6 translocates from the ER to the Golgi complex to be activated and then acts as a transcription factor after splicing^43,44^ As shown in Figure S3a,b, tunicamycin significantly upregulated Grp78 expression (transcriptional and translational) and key UPR mediators (Xbp11s, Atf4, Chop) in activating HSCs. The ER stress activation was also demonstrated by enhanced phosphorylation of the UPR-related regulator eIF2α (p-eiF2α) and ER expansion in activating HSCs (Figure S3b). Arctigenin treatment normalized the increased level of Grp78, and significantly attenuated UPR signaling through downregulation of Xbp1s, Atf4, and Chop expression (Figure S3a,b). This protective effect was further evidenced by the inhibition of p-eIF2α and mitigation of tunicamycin-induced ER expansion (Figure S3b). In activated HSCs, the ER stress reliever 4-PBA mimicked and increased the effect of arctigenin. 4-PBA alone mimicked arctigenin-mediated suppression of Grp78, Chop, and eIF2α phosphorylation, whereas their combined administration was even more effective in attenuating ER stress (Figure S3c,d). Both 4-PBA alone and its combination with arctigenin effectively restored the ER structure (Figure S3d). These findings collectively demonstrated the capacity of arctigenin to restore ER homeostasis by attenuating the UPR and restoring ER structure.

**Figure 3:**
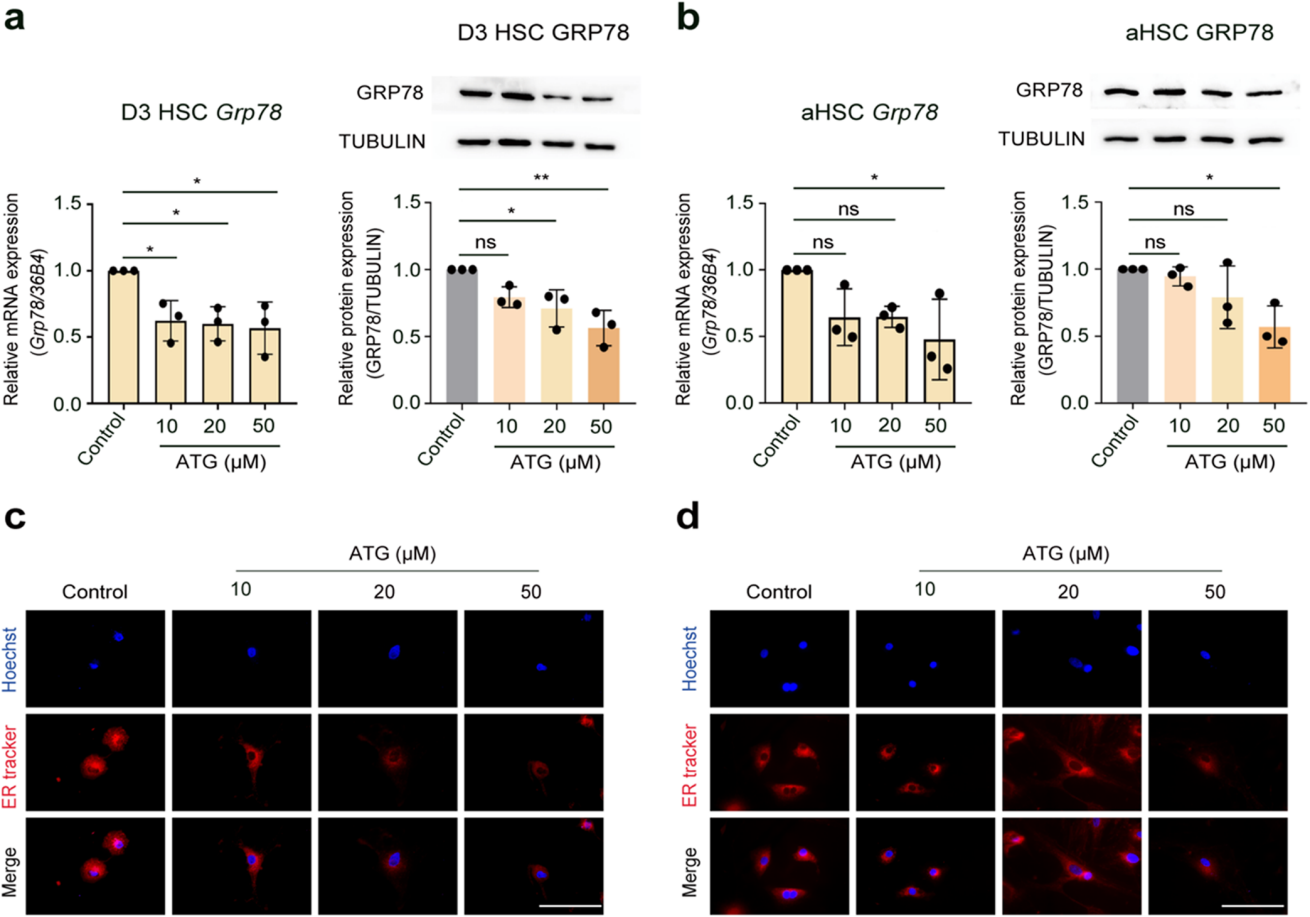
Arctigenin decreases ER stress initiation in hepatic stellate cells. Activating (Day 3) HSCs and activated (Day 7) HSCs (aHSCs) were treated with arctigenin (10, 20, and 50 μM) for 48 h. (a) Quantitative real-time PCR (qPCR) and Western blot analysis of mRNA and protein expression of the ER stress chaperone Grp78 in activating HSCs treated with arctigenin for 48 h. Protein intensity was analyzed by ImageJ software. (b) qPCR and Western blot analysis of Grp78 mRNA and protein expression in activated HSCs treated with arctigenin for 48 h, respectively. Protein intensity was analyzed by ImageJ software. (c) Live-cell ER-Tracker staining to assess ER abundance in activating HSCs treated with arctigenin for 48 h. Scale bar: 200 μm. (d) Live-cell ER-Tracker staining to evaluate ER abundance in activated HSCs treated with arctigenin for 48 h. Scale bar: 200 μm. HSC, Hepatic stellate cell; D3 HSC, activating HSCs; aHSC, D7 (fully) activated HSCs; ATG, arctigenin. Data are presented as mean ± standard deviation (mean ± SD, *n* = 3 per group). Statistical significance was determined by one-way ANOVA followed by Tukey’s post hoc test. Significance: ns: not significant, *P* > 0.05; *: *P* < 0.05; **: *P* < 0.01.

Further analysis uncovered the therapeutic potential of arctigenin in alleviating HSC activation *via* ER stress modulation: tunicamycin significantly increased the expression of HSC activation marker α-SMA in activating HSCs (Figure 4a–c), demonstrating that ER stress is a driver of HSC transdifferentiation and activation. Despite HSC activation, Col1a1 expression was significantly downregulated at both transcriptional and translational levels rather than upregulated following tunicamycin challenge (Figure 4a,b), which is consistent with a previous study demonstrating ER stress-mediated suppression of collagen synthesis.^45^ This phenomenon appears independent of transcriptional regulation of collagen biosynthetic enzymes (e.g., P4ha1 and P4ha2)^45^ but may be dependent on ER dysfunction-induced defects in post-translational processing of Col1α1, thereby resulting in the accumulation of misfolded Col1α1 in the ER lumen and depletion of secreted mature Col1α1.^45^ Both 4-PBA and arctigenin and their combination reduced both α-SMA and Col1a1 expression in activated HSCs (Figure 4d–f). Collectively, these data demonstrate that arctigenin suppresses HSC activation *via* alleviation of ER stress and restoration of ER homeostasis. Moreover, arctigenin significantly inhibits collagen biosynthesis, suggesting its potential to attenuate ECM remodeling in hepatic fibrosis. However, whether arctigenin directly targets collagen synthesis/secretion or acts indirectly by modulating ER expansion remains unclear. Further studies are required to clarify these mechanisms and the molecular targets involved.

**Figure 4:**
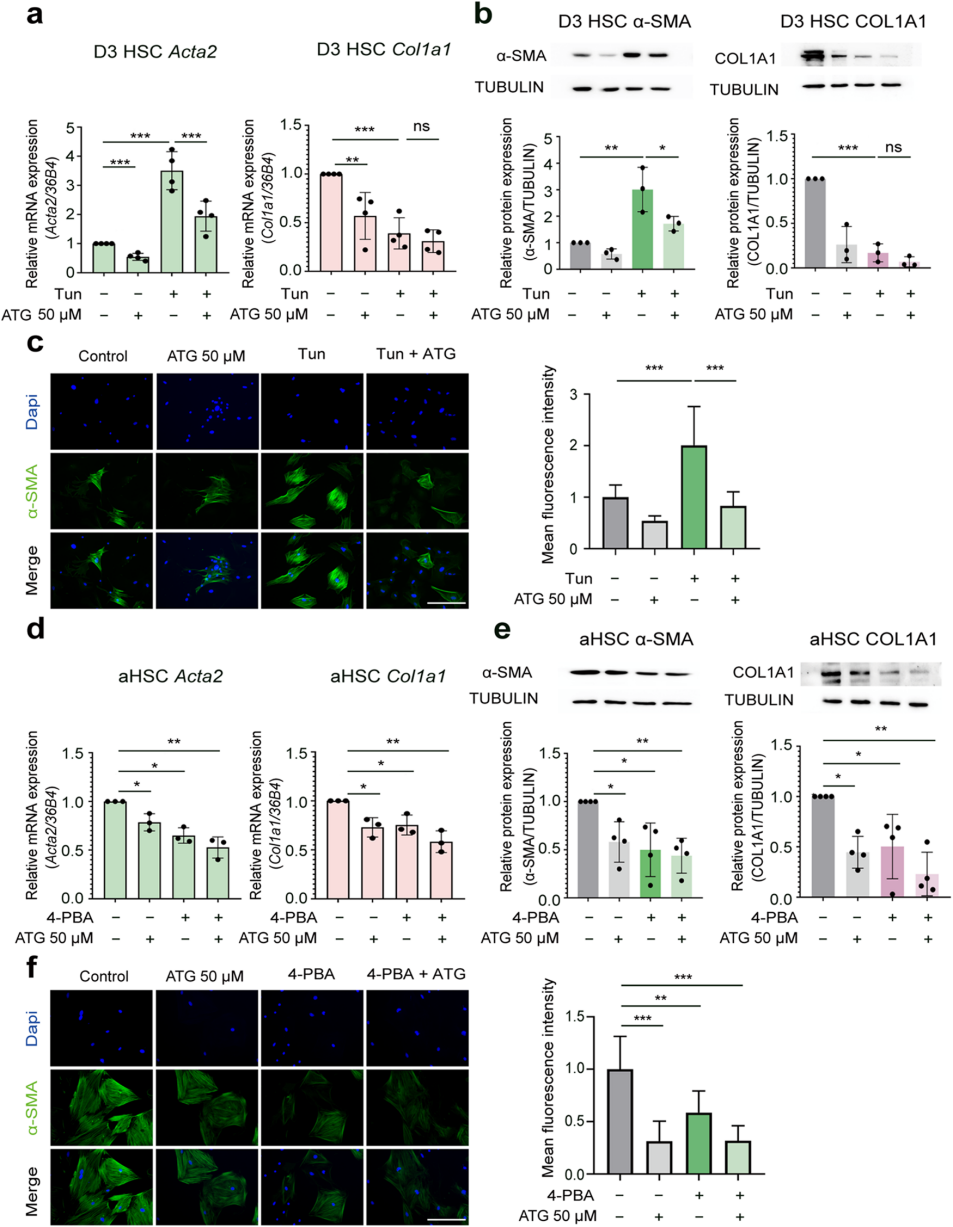
Arctigenin suppresses ER stress-induced hepatic stellate cell activation. Activating and activated HSCs were pretreated with arctigenin (50 μM, 12 h), followed by treatment with 2 μg/mL tunicamycin or 3 mM sodium 4-phenylbutyrate (4-PBA) for 12 h, respectively. (a) Quantitative real-time PCR (qPCR) analysis of mRNA expression of HSC activation marker α-smooth muscle actin (α-SMA) and extracellular matrix (ECM) molecule collagen type I α 1 (Col1α1) in activating HSCs. (b) Western blot analysis of α-SMA and Col1α1 protein expression in activating HSCs. Protein intensity was analyzed by ImageJ software. (c) Immunofluorescence analysis of α-SMA in activating HSCs. Fluorescence intensity was analyzed by ImageJ software. Scale bar: 200 μm. (d) qPCR analysis of α-SMA and Col1α1 mRNA expression in activated HSCs. (e) Western blot analysis of α-SMA and Col1α1 protein expression in activated HSCs. Protein intensity was analyzed by ImageJ software. (f) Immunofluorescence analysis of α-SMA in activated HSCs. Fluorescence Intensity was analyzed by ImageJ software. Scale bar: 200 μm. HSC, Hepatic stellate cell; D3 HSC, activating HSCs; aHSC, D7 (fully) activated HSCs; ATG, arctigenin; Tun, tunicamycin; 4-PBA, sodium 4-phenylbutyrate. Data are presented as mean ± standard deviation (mean ± SD, *n* ≥ 3 per group). Statistical significance was determined by one-way ANOVA followed by Tukey’s post hoc test. Significance: ns: not significant, *P* > 0.05; *: *P* < 0.05; **: *P* < 0.01; ***: *P* < 0.001.

### 3.3 Arctigenin Increases Neutral Rather Than Retinoid-Containing Lipid Droplets in ER Stress-Exposed Hepatic Stellate Cells

Lipid droplets (LDs), a characteristic feature of quiescent HSCs, serve as dynamic pools for retinoids and non-retinoid lipids.^26^ The loss of LDs and their lipid cargo is not only a hallmark of early HSC activation but is also hypothesized to supply bioenergetic substrates necessary for HSC activation.^3,46^ Consequently, maintaining LD integrity may represent a valid strategy to maintain HSC quiescence. ER dynamics, acting as a master regulator of LD homeostasis, orchestrates LD biogenesis: LD formation initiates within the ER bilayer and is dynamically controlled by lipogenic enzymes embedded in the ER membrane.^20,21,47^ Moreover, ER stress pathways critically regulate LD turnover, as indicated by studies demonstrating that UPR-driven autophagy promotes LD degradation.^44,48^ These findings position LDs as a central mechanism linking ER stress to HSC activation, although the precise regulatory crosstalk remains unresolved.

Since arctigenin has been shown to mitigate ER stress in HSCs (Figure S3), we hypothesized that arctigenin could reverse ER stress-mediated LD loss during HSC activation (experimental design shown in Figure S1c,d). To visualize retinoid-enriched LDs, we monitored alterations in vitamin A, the primary retinoid derivative, using its intrinsic fluorescence as a proxy for retinoid storage.^49^ Consistent with LD depletion during activation,^26^ *in vitro* HSC cultures exhibited a progressive decline in vitamin A autofluorescence (Figure S4a), reflecting retinoid loss during *in vitro* HSC activation. Arctigenin treatment significantly attenuated this reduction, preserving the LD-associated retinoid content. Retinoid homeostasis in HSCs is governed by a balance between storage (*via* re-esterification of retinol) and mobilization (*via* hydrolysis of retinyl esters).^50^ Lrat, the key enzyme catalyzing retinol esterification,^51^ was upregulated by arctigenin during HSC activation (Figure S4b), suggesting that arctigenin enhances re-esterification to preserve retinoid pools. We next investigated whether arctigenin restores retinoid-filled LDs by alleviating the ER stress. As shown in Figure 5a, tunicamycin triggered rapid retinoid loss in activating HSCs. However, arctigenin failed to rescue this depletion (Figure 5a). Transcriptional profiling revealed that tunicamycin significantly upregulated Pnpla3 and Cyp26a1, which drive retinol hydrolysis and metabolism,^52,53^ while suppressing Lrat expression (Figure 5b). Although arctigenin reduced Pnpla3 expression, it did not affect Lrat or Cyp26a1 levels in Tunicamycin-treated HSCs (Figure 5b). Collectively, these findings suggest that ER stress induces retinoid loss in HSCs *via* suppressed re-esterification (Lrat downregulation) and enhanced catabolism (Pnpla3/Cyp26a1 upregulation). Importantly, arctigenin attenuates ER stress-driven HSC activation through mechanisms independent of retinoid storage.

**Figure 5:**
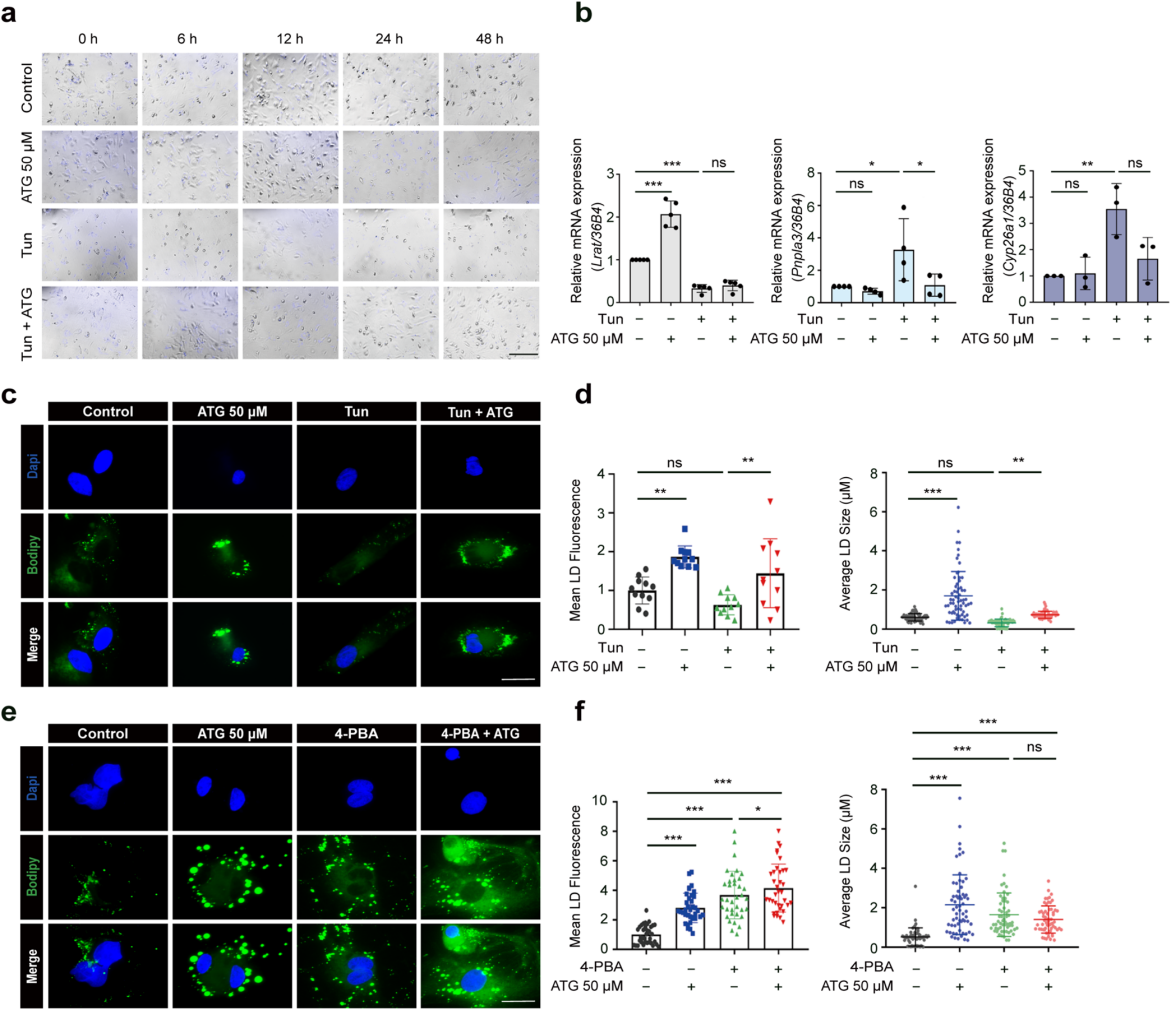
Arctigenin increases neutral but not retinoid lipid droplets in ER stress-exposed hepatic stellate cells. (a) Activating HSCs were pretreated with arctigenin (50 μM, 12 h), followed by treatment with 2 μg/mL tunicamycin for varying time intervals. Retinoid content within HSCs was visualized *via* intrinsic autofluorescence. Scale bar: 200 μm. (b–d) Activating HSCs were pretreated with arctigenin (50 μM, 12 h), followed by treatment with 2 μg/mL tunicamycin for 12 h. (b) Quantitative real-time PCR (qPCR) analysis of mRNA expression for genes involved in retinoid metabolism in activating HSCs. (c) BODIPY-LD staining of neutral lipid droplets (LDs) in activating HSCs. Scale bar: 25 μm. (d) Quantification of LD intensity and size in activating HSCs using ImageJ software. (e) and (f) Activated HSCs were pretreated with arctigenin (50 μM, 12 h), followed by treatment with 3 mM 4-phenylbutyric acid (4-PBA) for 12 h. (e) BODIPY-LD staining of neutral LDs in activated HSCs. Scale bar: 25 μm. (f) Quantification of LD intensity and size in activated HSCs using ImageJ software. HSC, Hepatic stellate cell; D3 HSC, activating HSCs; aHSC, D7 (fully) activated HSCs; ATG, arctigenin; Tun, tunicamycin; 4-PBA, sodium 4-phenylbutyrate. Data are presented as mean ± standard deviation (mean ± SD, *n* ≥ 3 per group). Statistical significance was determined by one-way ANOVA followed by Tukey’s post hoc test. Significance: ns: not significant, *P* > 0.05; *: *P* < 0.05; **: *P* < 0.01; ***: *P* < 0.001.

Neutral lipids, including triglycerides, cholesterol esters, non-esterified fatty acids, and diverse phospholipid species, constitute the predominant non-retinoid lipid components of LDs in HSCs.^26^ A key feature of HSC activation is the selective release of neutral lipids, particularly TGs, from LDs.^54^ Notably, pharmacological or genetic inhibition of lysosomal acid lipase LAL, a key enzyme driving triglyceride catabolism, attenuates HSC activation,^55,56^ underscoring the critical role of neutral lipid mobilization in driving fibrogenic signaling. To determine whether arctigenin modulates neutral lipid dynamics during ER stress, we visualized intracellular neutral LDs by using BODIPY staining. Interestingly, arctigenin supplementation not only restored neutral LD size but also increased fluorescence intensity in tunicamycin-treated activating HSCs (Figure 5c,d). Furthermore, attenuation of ER stress using 4-PBA, either alone or in combination with arctigenin, markedly increased both the intensity and the size of neutral LDs in activated HSCs (Figure 5e,f). These findings indicate that ER stress serves as a critical driver of neutral LD mobilization during HSC activation. Furthermore, arctigenin preserves neutral LD integrity by mitigating ER stress, which may suppress lipid-driven pro-fibrotic signaling.

It is critical to emphasize that our findings on LD dynamics under ER stress in HSCs differ from reports describing ER stress-induced lipid accumulation in hepatocytes.^57^ In hepatocytes, ER stress, even in the absence of lipid overload, promotes intracellular lipid retention through suppression of apolipoprotein ApoB100 expression and impaired lipoprotein secretion, resulting in hepatocyte steatosis.^57^ Conversely, HSCs, which inherently function as lipid reservoirs under physiological conditions, exhibit an ER stress-driven LD loss. Mechanistically, HSC activation downregulates apolipoprotein ApoE,^58,59^ a critical mediator of lipid secretion, while ER stress exacerbates lipid depletion by suppressing compensatory lipid deposition pathways in the absence of ApoE.^60^ These observations are consistent with prior evidence demonstrating that activated human HSCs treated with palmitate exhibit reduced lipid accumulation under high ER stress conditions,^61^ highlighting the unique capacity of HSCs to mobilize lipids in response to cellular stress, which is distinct from other hepatic cell types.

### 3.4 Arctigenin Modulates Lipid Droplet Size through Adaptive Lipogenesis and Lipolysis in ER Stress-Exposed Hepatic Stellate Cells

*De novo* lipogenesis (DNL) governs LD size by driving lipid biosynthesis and facilitating LD expansion through lipogenic enzymes, which catalyze triglyceride synthesis and mediate their deposition into the LD core.^20,47,62^ Under ER stress conditions, activation of the UPR suppresses lipogenesis by downregulating key enzymes, disrupting LD homeostasis.^63,64^ In tunicamycin-induced activating HSCs, we observed marked downregulation of Srebp1c (the transcriptional master regulator of fatty acid and triglyceride synthesis),^65^ and its downstream targets Acsl3 (essential for long-chain fatty acid activation) and Dgat2 (catalyzes the final step of triglyceride synthesis);^66,67^ however, enzymes involved in fatty acid elongation (Elovl5) or alternative lipid storage molecules (Dgat1, Ppar-γ) remained unaffected (Figure 6a).^68–70^ These findings indicate that ER stress selectively impairs the Srebp1c-driven DNL axis rather than globally disrupts lipid metabolism. Arctigenin treatment failed to restore Srebp1c, Acsl3, or Dgat2 expression but elevated Ppar-γ level (Figure 6a), suggesting that Srebp1c suppression under ER stress may involve irreversible transcriptional or epigenetic modifications, while Ppar-γ induction occurs *via* a distinct stress-responsive pathway. Furthermore, in activated HSCs, the ER stress alleviator 4-PBA did not alter the expression of Srebp1c, Dgat1, Acsl3, and Elovl5 but increased Dgat2 and Ppar-γ expression in the presence of arctigenin (Figure 6b). The partial restoration of Dgat2 implies that alleviating ER stress may ameliorate defects in lipid packaging, thereby promoting LD expansion through a Dgat2-dependent mechanism. Taken together, arctigenin may indirectly regulate lipid storage through enhancing the expression of compensatory molecules, i.e., Dgat2 and Ppar-γ, rather than restoring DNL in response to ER stress.

**Figure 6:**
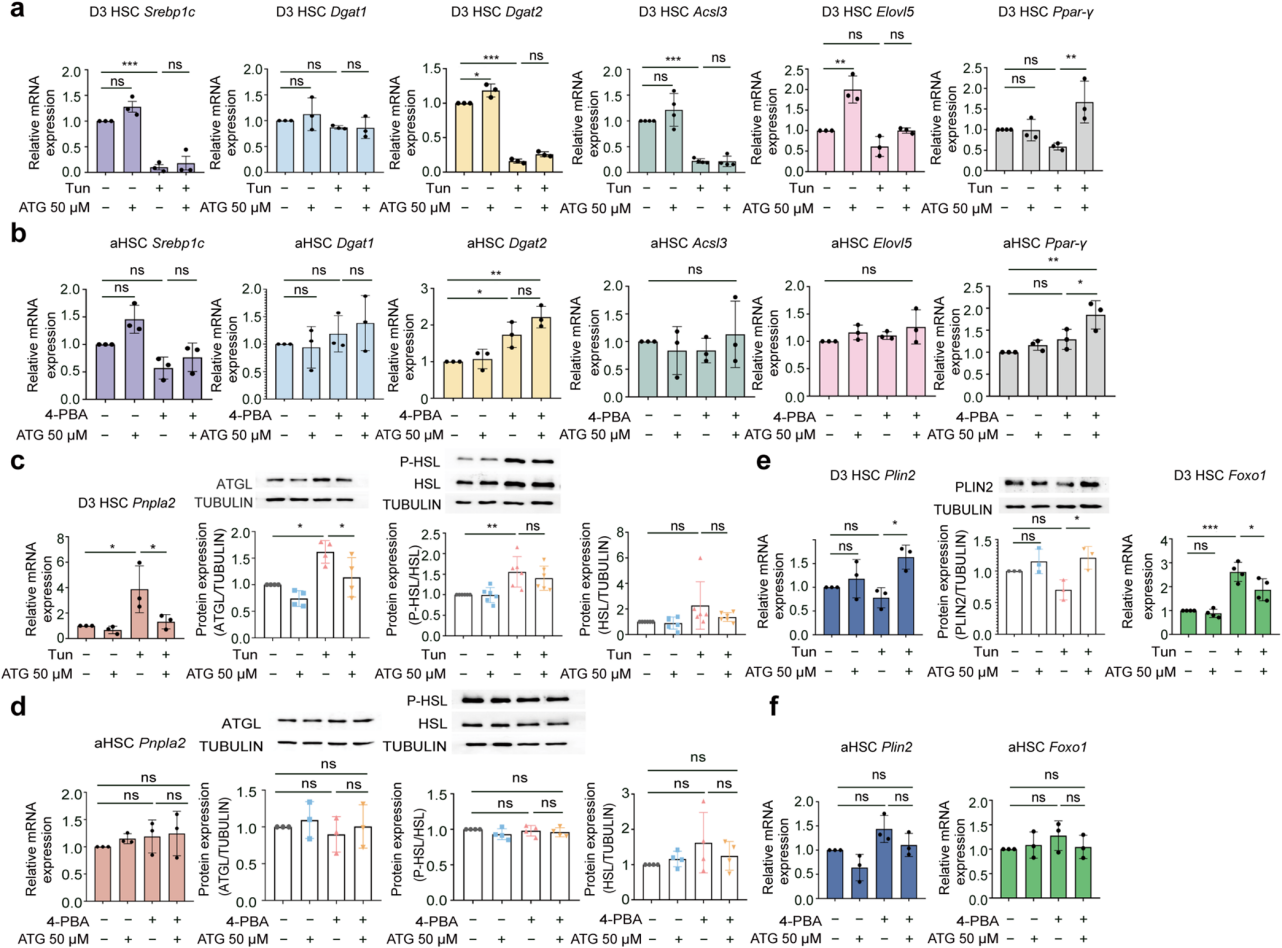
Arctigenin modulates lipogenesis and lipolysis in ER stress-exposed hepatic stellate cells. Activating and activated HSCs were first treated with arctigenin at 50 μM for 12 h followed by treatment with 2 μg/mL Tunicamycin or 3 mM 4-PBA for 12 h, respectively. (a) Quantitative real-time PCR (qPCR) analysis of mRNA expression for lipogenesis-related genes in activating HSCs. (b) qPCR analysis of lipogenesis-related gene mRNA expression in activated HSCs. (c) qPCR analysis of Pnpla2 (involved in lipolysis) mRNA expression and Western blot analysis of ATGL and HSL protein expression in activating HSCs. Protein intensity was analyzed by ImageJ software. (d) qPCR analysis of Pnpla2 mRNA expression and Western blot analysis of ATGL and HSL protein expression in activated HSCs. Protein intensity was analyzed by ImageJ software. (e) qPCR analysis of Plin2 and Foxo1 mRNA expression and Western blot analysis of PLIN2 protein expression in activating HSCs. Protein intensity was analyzed by ImageJ software. (f) qPCR analysis of Plin2 and Foxo1 mRNA expression in activated HSCs. HSC, Hepatic stellate cell; D3 HSC, activating HSCs; aHSC, D7 (fully) activated HSCs; ATG, arctigenin; Tun, tunicamycin; 4-PBA, sodium 4-phenylbutyrate. Data are presented as mean ± standard deviation (mean ± SD, *n* ≥ 3 per group). Statistical significance was determined by one-way ANOVA followed by Tukey’s post hoc test. Significance: ns: not significant, *P* > 0.05; *: *P* < 0.05; **: *P* < 0.01; ***: *P* < 0.001.

Lipolysis, the enzymatic hydrolysis of lipids within LDs, is another critical mechanism that regulates LD size apart from DNL.^71^ In contrast to DNL, ER stress actively enhances lipolysis by stimulating lipase-dependent pathways,^72–74^ thereby mobilizing stored lipids *via* lipases such as ATGL and HSL.^73^ In activating HSCs, tunicamycin-induced ER stress significantly upregulated both Pnpla2 (ATGL mRNA) and ATGL protein levels and increased phosphorylation of HSL (p-HSL, the active form) without altering total HSL expression (Figure 6c). This suggests that ER stress enhances lipolytic capacity through transcriptional (Pnpla2), translational (ATGL), and post-translational (HSL phosphorylation) mechanisms, likely as an adaptive response to mobilize fatty acids for energy production to facilitate HSC activation. Arctigenin treatment markedly reduced ATGL expression but did not affect p-HSL or total HSL (Figure 6c). This selective inhibition implies that arctigenin may interfere with transcriptional regulation of Pnpla2 or destabilize ATGL protein, while HSL phosphorylation is regulated independently of ATGL. Conversely, in activated HSCs, neither 4-PBA alone nor combined arctigenin/4-PBA altered ATGL or HSL expression (Figure 6d). This may be because expression of ATGL is already very low in activated HSCs and cannot be further reduced to maintain a minimal lipolytic activity necessary for cellular homeostasis.^75^ Hence, arctigenin may directly regulate ATGL-mediated lipolysis by suppressing its expression, thereby limiting LD degradation under the ER stress conditions.

Plin2 and Foxo1 function as regulators of ATGL-mediated lipolysis. Plin2, an LD-associated protein, colocalizes with Pnpla2 (the gene encoding ATGL) on LDs. Overexpression of Plin2 inhibits ATGL-dependent lipid hydrolysis, thus stabilizing LD content.^76^ Conversely, Foxo1, a transcription factor, directly activates Pnpla2 by binding to its promoter region, thereby enhancing triglyceride degradation.^77^ These opposing roles suggest that ER stress-induced changes in ATGL activity in HSCs may be mediated by Plin2 and Foxo1. As demonstrated in Figure 6e, tunicamycin-induced ER stress in activating HSCs did not alter Plin2 expression at either the mRNA or protein level, indicating that Plin2 is not directly involved in ER stress-driven ATGL activation. However, treatment with arctigenin significantly upregulated Plin2 levels, likely as a compensatory response to ATGL inhibition (Figure 6e). Conversely, tunicamycin drastically increased Foxo1 expression, which was abolished by arctigenin treatment (Figure 6e). This indicates that Foxo1 is a critical ER stress-sensitive transcriptional activator of ATGL, linking proteotoxic stress to enhanced lipolytic activity. In activated HSCs, neither the ER stress inhibitor 4-PBA nor arctigenin modulated Plin2 or Foxo1 levels (Figure 6f). This aligns with their lack of effect on ATGL in activated HSCs, suggesting that Plin2 and Foxo1 respond to ER stress in an activation-stage-dependent manner and exert their regulatory effects primarily during the early stress phase (activating HSCs) rather than the chronic phase (fully activated HSCs). Collectively, these findings indicate that Foxo1 is the primary ER stress-responsive regulator of ATGL, and its activity is regulated by the antilipolytic effect of arctigenin. Further mechanistic analyses are required to fully elucidate the precise role of Foxo1 in this pathway as well as its potential mechanism in the regulation of ATGL.

### 3.5 ERAD Is Involved in Arctigenin-Mediated Lipid Homeostasis in ER Stress-Exposed Hepatic Stellate Cells

The ERAD system serves as a critical protein quality control mechanism within the ER, functioning to preserve ER homeostasis by mediating the ubiquitin-dependent degradation of misfolded ER-resident proteins.^6^ Recent studies have further elucidated the pivotal role of ERAD in the regulation of lipid metabolism, where it governs the turnover of lipid biosynthetic enzymes *via* ubiquitination and subsequent proteasomal degradation, and enhances secretion of lipases.^19,25,78^ These findings suggest a potential relationship between the ERAD machinery and arctigenin-mediated lipid homeostasis, particularly under ER stress conditions. To investigate this hypothesis, we first assessed ERAD activity by analyzing the Syvn1 (Hrd1) ubiquitin ligase complex, a central regulatory branch of the mammalian ERAD system.^79^ ERAD substrates are recruited to the Syvn1 complex for degradation, facilitated by ER-resident cochaperones such as Dnajb9 or Dnajc10, which mediate substrate recognition. Following ubiquitination, substrates are transported to the cytosolic proteasome *via* the cytoplasmic adaptor Herpud1.^17,80^ As shown in Figure 7a, tunicamycin-induced ER stress significantly upregulated the expression of Dnajb9, Herpud1, and Syvn1 in activating HSCs. However, this upregulation was absent for Dnajc10 (Figure 7a), indicating that ERAD substrates are selectively recognized and interact with Dnajb9, but not Dnajc10, under these conditions, with Herpud1 mediating the delivery of ubiquitinated substrates to the proteasome, completing the ERAD cascade. Supplementation of arctigenin effectively downregulated the expression of Dnjb9, Herpud1, and Syvn1 in activating HSCs (Figure 7a), suggesting that arctigenin attenuates Syvn1-mediated ERAD activity during ER stress. Consistent with this, neither arctigenin nor the chemical chaperone 4-PBA altered Dnajc10 expression in activated HSCs (Figure 7b). Both arctigenin and 4-PBA reduced Dnajb9 and Herpud1 levels compared with untreated controls (Figure 7b). Although arctigenin alone or in combination with 4-PBA suppressed Syvn1 expression, 4-PBA alone had no effect on Syvn1 levels (Figure 7b). These results confirm that arctigenin inhibits Syvn1-ERAD activity at various stages of HSC activation by alleviating ER stress. Furthermore, the inability of 4-PBA to modulate Syvn1 implies that this ER stress mitigator may target alternative ERAD ubiquitin ligases, such as gp78, rather than Syvn1. Collectively, these findings demonstrate that the ERAD system is dynamically engaged in ER stress responses during HSC activation and modulated by arctigenin. This regulatory crosstalk indicates the potential role of ERAD in lipid homeostasis within the ER.

**Figure 7:**
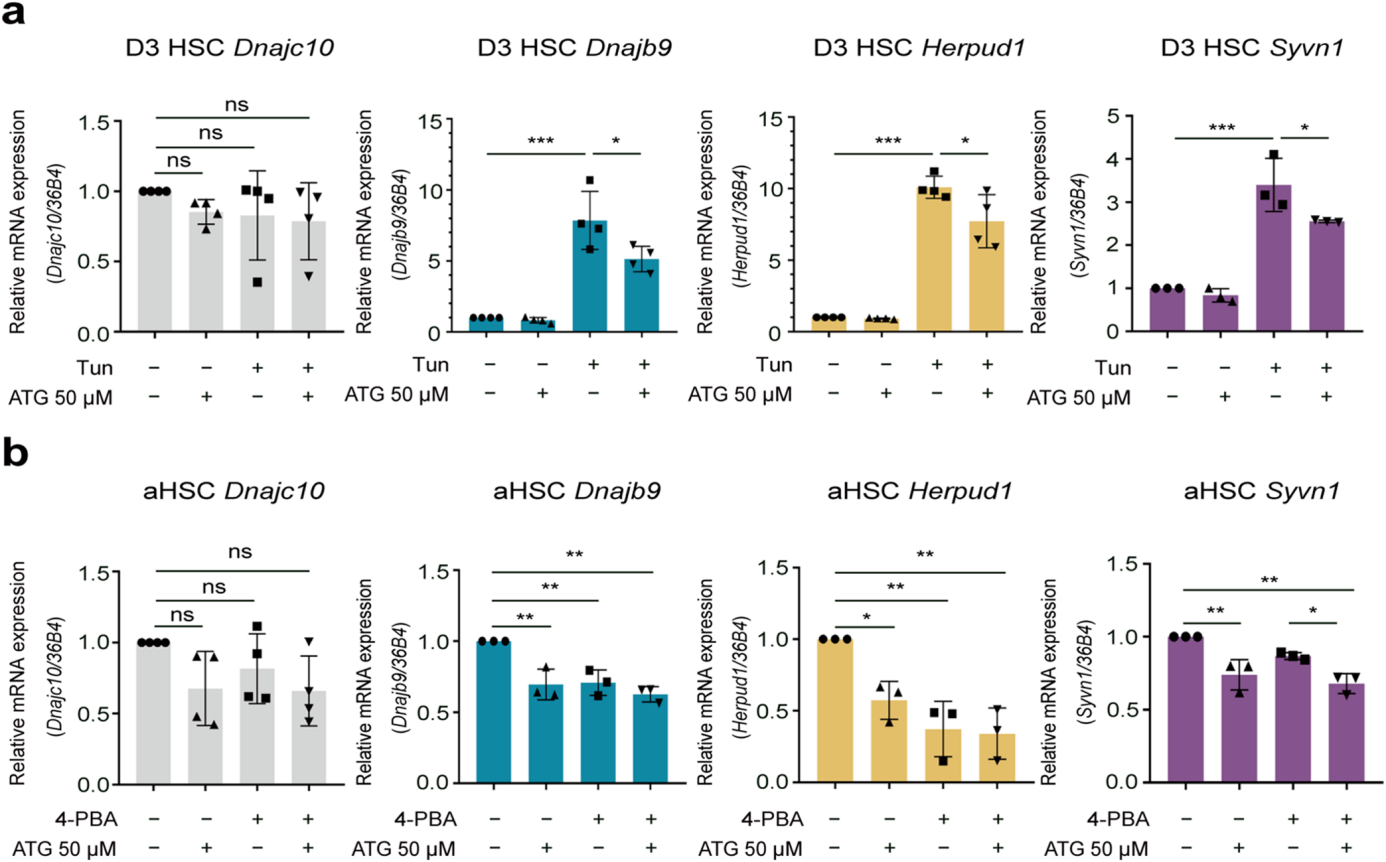
Arctigenin regulates ERAD homeostasis in ER stress-exposed hepatic stellate cells. Activating and activated HSCs were first treated with arctigenin at 50 μM for 12 h followed by treatment with 2 μg/mL Tunicamycin or 3 mM 4-PBA for 12 h, respectively. (a) Quantitative real-time PCR (qPCR) analysis of mRNA expression for genes associated with the ERAD system in activating HSCs. (b) qPCR analysis of ERAD-related gene mRNA expression in activated HSCs. HSC, Hepatic stellate cell; D3 HSC, activating HSCs; aHSC, D7 (fully) activated HSCs; ATG, arctigenin; Tun, tunicamycin; 4-PBA, sodium 4-phenylbutyrate. Data are presented as mean ± standard deviation (mean ± SD, *n* ≥ 3 per group). Statistical significance was determined by one-way ANOVA followed by Tukey’s post hoc test. Significance: ns: not significant, *P* > 0.05; *: *P* < 0.05; **: *P* < 0.01; ***: *P* < 0.001.

To further investigate the role of ERAD in the regulation of lipid homeostasis under ER stress, we treated ER stress-exposed HSCs with Eeyarestatin-I (Eer I), a potent ERAD inhibitor that selectively blocks deubiquitinating enzymes (experimental design shown in Figure S1e,f). As shown in Figure 8a,b, Eer I alone significantly reduced both mRNA and protein levels of the lipase ATGL (gene symbol Pnpla2) compared to tunicamycin-treated activating HSCs, even when co-administered with arctigenin. Eer I also downregulated the mRNA expression of Foxo1, an ER stress-responsive regulator of ATGL (Figure 6), in ER stress-induced activating HSCs. Notably, Eer I selectively increased the lipogenic gene Ppar-γ but not Dgat2 expression (Figure 8a). In activated HSCs, Eer I alone or combined with arctigenin markedly reduced ATGL protein (but not mRNA) levels compared with 4-PBA-treated activated HSCs (Figure 8c,d). Interestingly, Foxo1 mRNA levels remained unchanged by Eer I, even in combination with arctigenin (Figure 8c). Although Eer I alone or with arctigenin treatment did not alter the expression of lipogenic markers Dgat2 and Ppar-γ compared to 4-PBA-treated activated HSCs (Figure 8c), the unchanged Dgat2 levels may reflect a limitation to further upregulation following prior ER stress alleviation. These findings suggest that ERAD contributes to arctigenin-mediated lipid homeostasis through two mechanisms: suppression of adaptive lipogenesis and enhancement of ATGL-mediated lipolysis. ERAD inhibition upregulated the transcription level of adaptive lipogenic factor Ppar-γ and Dgat2, possibly due to impaired ubiquitin-proteasomal degradation of their upstream regulating (transcription) factors,^81,82^ implying that they may be indirectly regulated by ERAD modification. ERAD is essential for ATGL synthesis, likely *via* transcriptional (Foxo1-dependent) and post-translational mechanisms. In tunicamycin-treated HSCs (high ER stress), ERAD inhibition suppresses Foxo1 activity, thereby reducing the level of ATGL transcription. In 4-PBA-treated HSCs (low ER stress), ERAD directly stabilizes the ATGL protein. ERAD inhibition may induce immature ATGL retention within the ER lumen, triggering autophagic degradation or impairing its translocation to ER.^25,83^ Further studies are needed to identify ERAD-targeted lipogenic factors as well as the mechanism by which ERAD stabilizes ATGL, to fully elucidate ERAD as a key mediator of arctigenin-mediated LD homeostasis.

**Figure 8:**
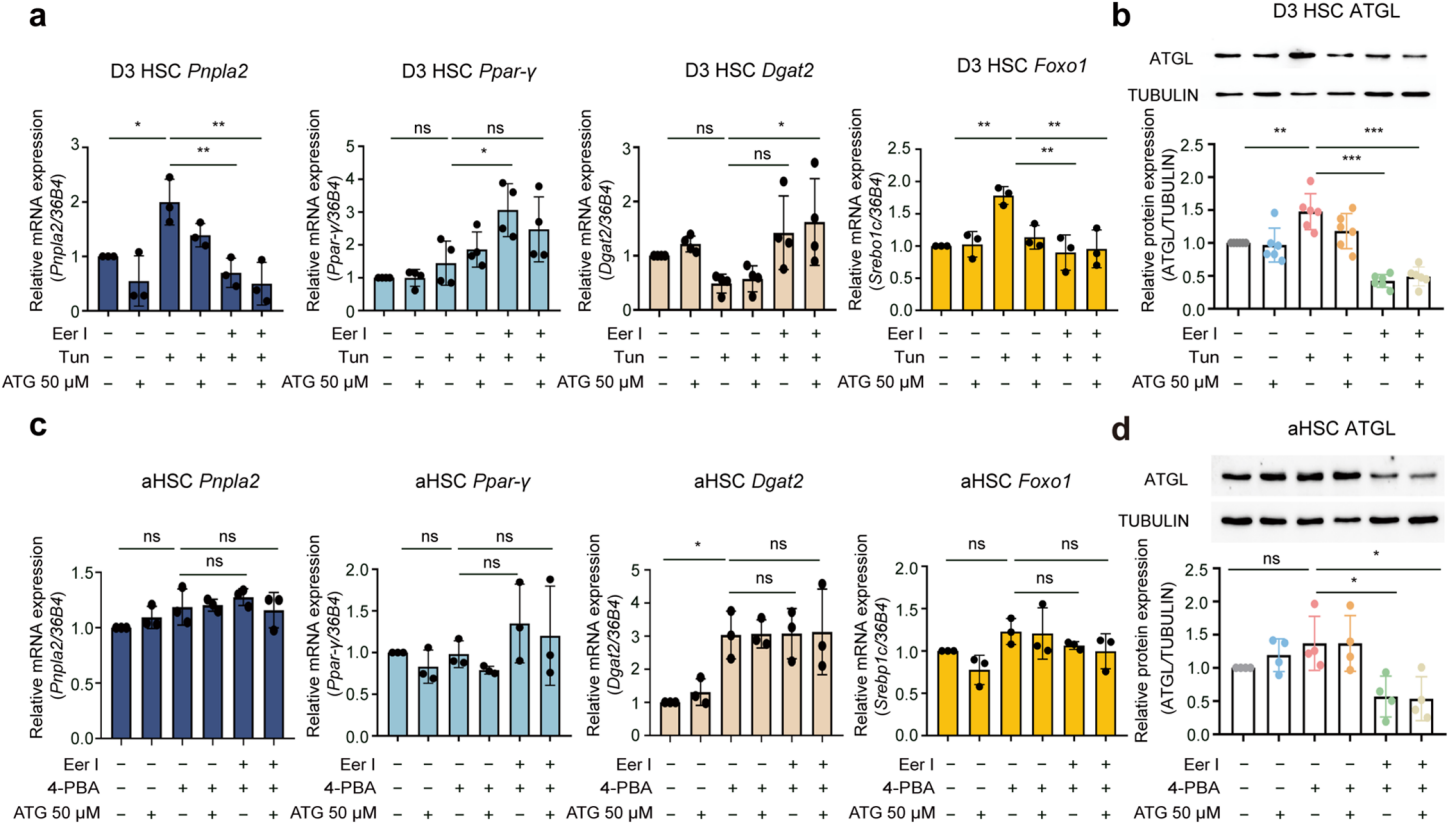
ERAD is involved in arctigenin-mediated lipid homeostasis in ER stress-exposed hepatic stellate cells. (a) Activating HSCs were first treated with arctigenin at 50 μM for 12 h followed by treatment with 2 μg/mL tunicamycin alone or in combination with 2 μM Eer I for 8 h. Real-time PCR analysis of mRNA expression of genes involved in lipolysis and lipogenesis in activating HSCs. (b) Activating HSCs were first treated with arctigenin at 50 μM for 12 h followed by treatment with 2 μg/mL tunicamycin alone or in combination with 2 μM Eer I for 10 h. Western blot analysis of protein expression of Pnpla2 in activating HSCs. Protein intensity was analyzed by ImageJ software. (c) Activated HSCs were first treated with arctigenin at 50 μM for 12 h followed by treatment with 3 mM 4-PBA alone or in combination with 2 μM Eer I for 8 h. Real-time PCR analysis of mRNA expression of genes involved in lipolysis and lipogenesis in activated HSCs. (d) Activated HSCs were first treated with arctigenin at 50 μM for 12 h followed by treatment with 3 mM 4-PBA alone or in combination with 2 μM Eer I for 10 h. Western blot analysis of protein expression of Pnpla2 in activated HSCs. Protein intensity was analyzed by ImageJ software. HSC, Hepatic stellate cell; D3 HSC, activating HSCs; aHSC, D7 (fully) activated HSCs; ATG, arctigenin; Tun, tunicamycin; 4-PBA, sodium 4-phenylbutyrate; Eer I, Eeyarestatin-I. Data are presented as mean ± standard deviation (mean ± SD, *n* ≥ 3 per group). Statistical significance was determined by one-way ANOVA followed by Tukey’s post hoc test. Significance: ns: not significant, *P* > 0.05; *: *P* < 0.05; **: *P* < 0.01; ***: *P* < 0.001.

In summary, our study elucidates a previously unrecognized role and mechanistic pathway of arctigenin, a bioactive lignan derived from Arctium lappa L., in mitigating ER stress-driven HSC activation. We demonstrate that arctigenin suppresses HSC activation by restoring lipid homeostasis through ERAD-dependent mechanisms under ER stress. ER stress induces lipid dysregulation, characterized by the loss of LDs, which is mechanistically dependent on ERAD activation through modulating the imbalance of adaptive lipogenesis and lipolysis. Arctigenin alleviates these effects by normalizing ERAD activity, thereby preserving LD abundance and size through dual modulation, upregulating lipogenic factors (Ppar-γ, Dgat2) and suppressing lipolytic drivers (ATGL). A critical limitation of this work is its exclusive reliance on *in vitro* models, which constrains translational applicability. To advance arctigenin toward clinical practice or food supplements, future studies should validate these findings *in vivo* using animal models of liver fibrosis to assess the efficacy, pharmacokinetics, and safety of arctigenin. Furthermore, the molecular targets of arctigenin within the ERAD cascade remain unclear, warranting proteomic or structural studies. Investigating the synergistic effects of arctigenin with existing antifibrotics and optimizing its bioavailability as a nutraceutical agent are crucial for clinical translation. Addressing these gaps will enhance understanding of the therapeutic potential of arctigenin and ER stress-lipid interplay in fibrotic diseases.

## Supplementary Material



## Notes

Schematic flowchart illustrating experimental design (Figure S1); determination of optimal concentrations of arctigenin (Figure S2); arctigenin inhibits the UPR in ER stress-exposed hepatic stellate cells (Figure S3); and arctigenin increases retinoid content in activating hepatic stellate cells (Figure S4) (PDF)

## Abbreviations

Acsl3: acyl-CoA synthetase long-chain family member 3
Acta2: actin α 2, smooth muscle
α-SMA: α-smooth muscle actin
Atf4: activating transcription factor 4
ATGL: adipose triglyceride lipase
Chop: C/EBP homologous protein
Col1a1: collagen type I α 1 chain
Cyp26a1: cytochrome P450 family 26 subfamily A member 1
Dgat1: diacylglycerol O-acyltransferase 1
Dgat2: diacylglycerol O-acyltransferase 2
Danjb9: DnaJ heat shock protein family (Hsp40) member B9
Dnajc10: DnaJ heat shock protein family (Hsp40) member C10
Eer I: eeyarestatin-I
eIF2α: eukaryotic initiation factor 2
Elovl5: ELOVL fatty acid elongase 5
ER: endoplasmic reticulum
ERAD: endoplasmic reticulum-associated degradation
Foxo1: Forkhead box protein O1
Grp78: glucose-regulated protein 78
Herpud1: homocysteine inducible ER protein with ubiquitin like domain 1
HSC: hepatic stellate cell
LD: lipid droplet
Lrat: lecithin retinol acyltransferase
4-PBA: sodium 4-phenylbutyrate
Plin2: perilipin 2
Pnpla2: patatin like phospholipase domain containing 2
Pnpla3: patatin like phospholipase domain containing 3
Ppar-γ: peroxisome proliferator-activated receptor γ
Srebp1c: sterol regulatory element binding protein 1c
Syvn1: synoviolin 1
UPR: unfolded protein response
Xbp1s: spliced form of X-box binding protein 1

## References

[1] Bataller R., Brenner D. A. (2005). Liver fibrosis. J. Clin. Invest..

[2] Younossi Z.
M. (2019). Non-alcoholic
fatty liver disease—A global public health perspective. J. Hepatol..

[3] Tsuchida T., Friedman S. L. (2017). Mechanisms of hepatic stellate cell
activation. Nat. Rev. Gastroenterol. Hepatol..

[4] Khomich O., Ivanov A. V., Bartosch B. (2020). Metabolic hallmarks of hepatic stellate
cells in liver fibrosis. Cells.

[5] Chan Y. T., Wang N., Tan H. Y., Li S., Feng Y. (2020). Targeting
Hepatic Stellate Cells for the Treatment of Liver Fibrosis by Natural
Products: Is It the Dawning of a New Era?. Front.
Pharmacol..

[6] Hwang J., Qi L. (2018). Quality Control in
the Endoplasmic Reticulum: Crosstalk between ERAD
and UPR pathways. Trends Biochem. Sci..

[7] Chen X., Shi C., He M., Xiong S., Xia X. (2023). Endoplasmic reticulum
stress: molecular mechanism and therapeutic targets. Signal Transduction Targeted Ther..

[8] Koo J. H., Lee H. J., Kim W., Kim S. G. (2016). Endoplasmic Reticulum
Stress in Hepatic Stellate Cells Promotes Liver Fibrosis via PERK-Mediated
Degradation of HNRNPA1 and Up-regulation of SMAD2. Gastroenterology.

[9] Choi J. A., Song C. H. (2020). Insights Into the
Role of Endoplasmic Reticulum Stress
in Infectious Diseases. Front. Immunol..

[10] Marciniak S. J., Chambers J. E., Ron D. (2022). Pharmacological
targeting of endoplasmic
reticulum stress in disease. Nat. Rev. Drug
Discovery.

[11] Kim R. S., Hasegawa D., Goossens N., Tsuchida T., Athwal V., Sun X., Robinson C. L., Bhattacharya D., Chou H. I., Zhang D. Y., Fuchs B. C., Lee Y., Hoshida Y., Friedman S. L. (2016). The XBP1
Arm of the Unfolded Protein Response Induces Fibrogenic Activity in
Hepatic Stellate Cells Through Autophagy. Sci.
Rep..

[12] De
Minicis S., Candelaresi C., Agostinelli L., Taffetani S., Saccomanno S., Rychlicki C., Trozzi L., Marzioni M., Benedetti A., Svegliati-Baroni G. (2012). Endoplasmic Reticulum stress induces hepatic stellate
cell apoptosis and contributes to fibrosis resolution. Liver Int..

[13] Pavlović N., Heindryckx F. (2022). Targeting
ER stress in the hepatic tumor microenvironment. FEBS J..

[14] de
Galarreta M. R., Navarro A., Ansorena E., Garzón A. G., Mòdol T., López-Zabalza M. J., Martínez-Irujo J. J., Iraburu M. J. (2016). Unfolded protein response induced by Brefeldin A increases
collagen type I levels in hepatic stellate cells through an IRE1α,
p38 MAPK and Smad-dependent pathway. Biochim.
Biophys. Acta, Mol. Cell Res..

[15] Hernández-Gea V., Hilscher M., Rozenfeld R., Lim M. P., Nieto N., Werner S., Devi L. A., Friedman S. L. (2013). Endoplasmic reticulum
stress induces fibrogenic activity in hepatic stellate cells through
autophagy. J. Hepatol..

[16] Ma Z., Sheng L., Li J., Qian J., Wu G., Wang Z., Zhang Y. (2022). Resveratrol
Alleviates Hepatic Fibrosis
in Associated with Decreased Endoplasmic Reticulum Stress-Mediated
Apoptosis and Inflammation. Inflammation.

[17] Krshnan L., van de Weijer M. L., Carvalho P. (2022). Endoplasmic Reticulum–Associated
Protein Degradation. Cold Spring Harbor Perspect.
Biol..

[18] Hasegawa D., Fujii R., Yagishita N., Matsumoto N., Aratani S., Izumi T., Azakami K., Nakazawa M., Fujita H., Sato T., Araya N., Koike J., Tadokoro M., Suzuki N., Nagata K., Senoo H., Friedman S. L., Nishioka K., Yamano Y., Itoh F., Nakajima T. (2010). E3 ubiquitin ligase synoviolin is
involved in liver
fibrogenesis. PLoS One.

[19] Stevenson J., Huang E. Y., Olzmann J. A. (2016). Endoplasmic
Reticulum-Associated
Degradation and Lipid Homeostasis. Annu. Rev.
Nutr..

[20] Zadoorian A., Du X., Yang H. (2023). Lipid droplet biogenesis and functions in health and
disease. Nat. Rev. Endocrinol..

[21] Walther T. C., Chung J., Farese R. V. (2017). Lipid droplet
biogenesis. Annu. Rev. Cell Dev. Biol..

[22] Rajakumar S., Vijayakumar R., Abhishek A., Selvam G. S., Nachiappan V. (2020). Loss of ERAD
bridging factor UBX2 modulates lipid metabolism and leads to ER stress-associated
apoptosis during cadmium toxicity in Saccharomyces cerevisiae. Curr. Genet..

[23] Lee J. N., Kim H., Yao H., Chen Y., Weng K., Ye J. (2010). Identification
of Ubxd8 protein as a sensor for unsaturated fatty acids and regulator
of triglyceride synthesis. Proc. Natl. Acad.
Sci. U.S.A..

[24] Luo H., Jiang M., Lian G., Liu Q., Shi M., Li T. Y., Song L., Ye J., He Y., Yao L., Zhang C., Lin Z. Z., Zhang C. S., Zhao T. J., Jia W. P., Li P., Lin S. Y., Lin S. C. (2018). AIDA Selectively
Mediates Downregulation of Fat Synthesis Enzymes by ERAD to Retard
Intestinal Fat Absorption and Prevent Obesity. Cell Metab..

[25] Sha H., Sun S., Francisco A. B., Ehrhardt N., Xue Z., Liu L., Lawrence P., Mattijssen F., Guber R. D., Panhwar M. S., Brenna J. T., Shi H., Xue B., Kersten S., Bensadoun A., Péterfy M., Long Q., Qi L. (2014). The ER-associated
degradation adaptor protein sel1l regulates LPL secretion and lipid
metabolism. Cell Metab..

[26] Shmarakov I. O., Jiang H., Liu J., Fernandez E. J., Blaner W. S. (2019). Hepatic stellate cell activation:
A source for bioactive
lipids. Biochim. Biophys. Acta, Mol. Cell Biol.
Lipids.

[27] Shukla S., Kakade M., Cherian S., Alagarasu K., Parashar D. (2024). Arctigenin from Arctium lappa L. inhibits chikungunya virus by affecting its entry and replication. Phytomedicine.

[28] Gu Y., Sun X. X., Ye J. M., He L., Yan S. S., Zhang H. H., Hu L. H., Yuan J. Y., Yu Q. (2012). Arctigenin
alleviates ER stress via activating AMPK. Acta
Pharmacol. Sin..

[29] Zhang J., Cao P., Gui J., Wang X., Han J., Wang Y., Wang G. (2019). Arctigenin ameliorates renal impairment and inhibits endoplasmic
reticulum stress in diabetic db/db mice. Life
Sci..

[30] Kim J. Y., Hwang J. H., Cha M. R., Yoon M. Y., Son E. S., Tomida A., Ko B., Song S. W., Shin-Ya K., Hwang Y. Il., Park H. R. (2010). Arctigenin
blocks the unfolded protein
response and shows therapeutic antitumor activity. J. Cell. Physiol..

[31] Moshage H., Casini A., Lieber C. S. (1990). Acetaldehyde
selectively stimulates
collagen production in cultured rat liver fat-storing cells but not
in hepatocytes. Hepatology.

[32] Tomita K., Teratani T., Suzuki T., Shimizu M., Sato H., Narimatsu K., Okada Y., Kurihara C., Irie R., Yokoyama H., Shimamura K., Usui S., Ebinuma H., Saito H., Watanabe C., Komoto S., Kawaguchi A., Nagao S., Sugiyama K., Hokari R., Kanai T., Miura S., Hibim T. (2014). Free cholesterol
accumulation in
hepatic stellate cells: Mechanism of liver fibrosis aggravation in
nonalcoholic steatohepatitis in mice. Hepatology.

[33] Xia M., Wu Z., Wang J., Buist-Homan M., Moshage H. (2023). The Coumarin-Derivative
Esculetin Protects against Lipotoxicity in Primary Rat Hepatocytes
via Attenuating JNK-Mediated Oxidative Stress and Attenuates Free
Fatty Acid-Induced Lipid Accumulation. Antioxidants.

[34] Lv M., Chen S., Shan M., Si Y., Huang C., Chen J., Gong L. (2024). Arctigenin induces
activated HSCs
quiescence via AMPK-PPARγ pathway to ameliorate liver fibrosis
in mice. Eur. J. Pharmacol..

[35] Lebaron P., Catala P., Parthuisot N. (1998). Effectiveness
of SYTOX green stain
for bacterial viability assessment. Appl. Environ.
Microbiol..

[36] Madorran E., Ambrož M., Knez J., Sobočan M. (2025). An Overview
of the Current State of Cell Viability Assessment Methods Using OECD
Classification. Int. J. Mol. Sci..

[37] Wells R. G. (2008). Cellular
Sources of Extracellular Matrix in Hepatic Fibrosis. Clin. Liver Dis..

[38] Ozdemir A., Ark M. (2014). xCELLigence Real Time
Cell Analysis System: A New Method for Cell
Proliferation and Cytotoxicity. Niche J..

[39] Gómez-Lechón M. J., O’Connor J. E., Lahoz A., Castell J. V., Donato M. T. (2008). Identification
of Apoptotic Drugs: Multiparametric Evaluation in Cultured Hepatocytes. Curr. Med. Chem..

[40] Pellarin I., Dall’Acqua A., Favero A., Segatto I., Rossi V., Crestan N., Karimbayli J., Belletti B., Baldassarre G. (2025). Cyclin-dependent
protein kinases and cell cycle regulation in biology and disease. Signal Transduction Targeted Ther..

[41] Li A., Wang J., Wu M., Zhang X., Zhang H. (2015). The inhibition
of activated hepatic stellate cells proliferation by arctigenin through
G0/G1 phase cell cycle arrest: Persistent p27Kip1 induction by interfering
with PI3K/Akt/FOXO3a signaling pathway. Eur.
J. Pharmacol..

[42] Weiskirchen R., Weimer J., Meurer S. K., Kron A., Seipel B., Vater I., Arnold N., Siebert R., Xu L., Friedman S. L., Bergmann C. (2013). Genetic Characteristics
of the Human
Hepatic Stellate Cell Line LX-2. PLoS One.

[43] Spencer B. G., Finnie J. W. (2020). The Role of Endoplasmic Reticulum Stress in Cell Survival
and Death. J. Comp. Pathol..

[44] Hanquier Z., Misra J., Baxter R., Maiers J. L. (2023). Stress and Liver
Fibrogenesis: Understanding the Role and Regulation of Stress Response
Pathways in Hepatic Stellate Cells. Am. J. Pathol..

[45] Vonk L. A., Doulabi B. Z., Huang C. L., Helder M. N., Everts V., Bank R. A. (2010). Endoplasmic reticulum stress inhibits collagen synthesis
independent of collagen-modifying enzymes in different chondrocyte
populations and dermal fibroblasts. Biochem.
Cell Biol..

[46] Horn P., Tacke F. (2024). Metabolic reprogramming in liver fibrosis. Cell Metab..

[47] Olzmann J. A., Carvalho P. (2019). Dynamics and functions of lipid droplets. Nat. Rev. Mol. Cell Biol..

[48] Hernndezgea V., Ghiassinejad Z., Rozenfeld R., Gordon R., Fiel M. I., Yue Z., Czaja M. J., Friedman S. L. (2012). Autophagy releases lipid that promotes
fibrogenesis by activated hepatic stellate cells in mice and in human
tissues. Gastroenterology.

[49] Saeed A., Bartuzi P., Heegsma J., Dekker D., Kloosterhuis N., de Bruin A., Jonker J. W., van de Sluis B., Faber K. N. (2021). Impaired Hepatic Vitamin A Metabolism
in NAFLD Mice
Leading to Vitamin A Accumulation in Hepatocytes. Cell. Mol. Gastroenterol. Hepatol..

[50] Ghyselinck N. B., Båvik C., Sapin V., Mark M., Bonnier D., Hindelang C., Dierich A., Nilsson C. B., Håkansson H., Sauvant P., Azaïs-Braesco V., Frasson M., Picaud S., Chambon P. (1999). Cellular retinol-binding
protein
I is essential for vitamin A homeostasis. EMBO
J..

[51] Sears A. E., Palczewski K. (2016). Lecithin:Retinol Acyltransferase:
A Key Enzyme Involved
in the Retinoid (visual) Cycle. Biochemistry..

[52] Pirazzi C., Valenti L., Motta B. M., Pingitore P., Hedfalk K., Mancina R. M., Burza M. A., Indiveri C., Ferro Y., Montalcini T., Maglio C., Dongiovanni P., Fargion S., Rametta R., Pujia A., Andersson L., Ghosal S., Levin M., Wiklund O., Iacovino M., Borén J., Romeo S. (2014). PNPLA3 has retinyl-palmitate lipase
activity in human hepatic stellate cells. Hum.
Mol. Genet..

[53] Thatcher J. E., Isoherranen N. (2009). The role of CYP26 enzymes in retinoic acid clearance. Expert Opin. Drug Metab. Toxicol..

[54] Molenaar M. R., Vaandrager A. B., Helms J. B. (2017). Some lipid droplets are more equal
than others: Different metabolic lipid droplet pools in hepatic stellate
cells. Lipid Insights.

[55] Tuohetahuntila M., Molenaar M. R., Spee B., Brouwers J. F., Wubbolts R., Houweling M., Yan C., Du H., VanderVen B. C., Vaandrager A. B., Helms J. B. (2017). Lysosome-mediated
degradation of
a distinct pool of lipid droplets during hepatic stellate cell activation. J. Biol. Chem..

[56] Trivedi P., Wang S., Friedman S. L. (2021). The Power
of Plasticity—Metabolic
Regulation of Hepatic Stellate Cells. Cell Metab..

[57] Feng B., Huang X., Jiang D., Hua L., Zhuo Y., Wu D. (2017). Endoplasmic reticulum stress inducer tunicamycin alters hepatic energy
homeostasis in mice. Int. J. Mol. Sci..

[58] Schinagl M., Tomin T., Gindlhuber J., Honeder S., Pfleger R., Schittmayer M., Trauner M., Birner-Gruenberger R. (2021). Proteomic
changes of activated hepatic stellate cells. Int. J. Mol. Sci..

[59] Friedman S. L. (2008). Hepatic
stellate cells: Protean, multifunctional, and enigmatic cells of the
liver. Physiol. Rev..

[60] Ma M., Song L., Yan H., Liu M., Zhang L., Ma Y., Yuan J., Hu J., Ji Z., Zhang R., Li C., Wang H., Tao L., Zhang Y., Li Y. (2016). Low dose tunicamycin
enhances atherosclerotic plaque stability by inducing autophagy. Biochem. Pharmacol..

[61] Hetherington A. M., Sawyez C. G., Zilberman E., Stoianov A. M., Robson D. L., Borradaile N. M. (2016). Differential
Lipotoxic Effects of Palmitate and Oleate
in Activated Human Hepatic Stellate Cells and Epithelial Hepatoma
Cells. Cell. Physiol. Biochem..

[62] Yu J., Li P. (2017). The size matters: regulation
of lipid storage by lipid droplet dynamics. Sci. China: Life Sci..

[63] Celik C., Lee S. Y. T., Yap W. S., Thibault G. (2023). Endoplasmic reticulum
stress and lipids in health and diseases. Prog.
Lipid Res..

[64] Rutkowski D. T., Wu J., Back S. H., Callaghan M. U., Ferris S. P., Iqbal J., Clark R., Miao H., Hassler J. R., Fornek J., Katze M. G., Hussain M. M., Song B., Swathirajan J., Wang J., Yau G. D., Kaufman R. J. (2008). UPR Pathways Combine
to Prevent Hepatic Steatosis Caused by ER Stress-Mediated Suppression
of Transcriptional Master Regulators. Dev. Cell..

[65] Eberlé D., Hegarty B., Bossard P., Ferré P., Foufelle F. (2004). SREBP transcription factors: Master regulators of lipid
homeostasis. Biochimie.

[66] Klasson T. D., LaGory E. L., Zhao H., Huynh S. K., Papandreou I., Moon E. J., Giaccia A. J. (2022). ACSL3 regulates
lipid droplet biogenesis
and ferroptosis sensitivity in clear cell renal cell carcinoma. Cancer Metab..

[67] McLaren D. G., Han S., Murphy B. A., Wilsie L., Stout S. J., Zhou H., Roddy T. P., Gorski J. N., Metzger D. E., Shin M. K., Reilly D. F., Zhou H. H., Tadin-Strapps M., Bartz S. R., Cumiskey A. M., Graham T. H., Shen D. M., Akinsanya K. O., Previs S. F., Imbriglio J. E., Pinto S. (2018). DGAT2 Inhibition Alters Aspects of Triglyceride Metabolism in Rodents
but Not in Non-human Primates. Cell Metab..

[68] Wang Y., Torres-Gonzalez M., Tripathy S., Botolin D., Christian B., Jump D. B. (2008). Elevated hepatic fatty acid elongase-5 activity affects
multiple pathways controlling hepatic lipid and carbohydrate composition. J. Lipid Res..

[69] McLelland G. L., Lopez-Osias M., Verzijl C. R. C., Ellenbroek B. D., Oliveira R. A., Boon N. J., Dekker M., van den
Hengel L. G., Ali R., Janssen H., Song J. Y., Krimpenfort P., van Zutphen T., Jonker J. W., Brummelkamp T. R. (2023). Identification
of an alternative triglyceride biosynthesis pathway. Nature.

[70] Gorga A., Rindone G. M., Regueira M., Pellizzari E. H., Camberos M. C., Cigorraga S. B., Riera M. F., Galardo M. N., Meroni S. B. (2017). PPARγ activation
regulates lipid droplet formation
and lactate production in rat Sertoli cells. Cell Tissue Res..

[71] Suzuki M., Shinohara Y., Ohsaki Y., Fujimoto T. (2011). Lipid droplets:
Size
matters. J. Electron Microsc..

[72] Miyoshi H., Perfield J. W., Obin M. S., Greenberg A. S. (2008). Adipose
triglyceride lipase regulates basal lipolysis and lipid droplet size
in adipocytes. J. Cell. Biochem..

[73] Deng J., Liu S., Zou L., Xu C., Geng B., Xu G. (2012). Lipolysis
response to endoplasmic reticulum stress in adipose cells. J. Biol. Chem..

[74] Mao H. Z., Ehrhardt N., Bedoya C., Gomez J. A., DeZwaan-McCabe D., Mungrue I. N., Kaufman R. J., Rutkowski D. T., Péterfy M. (2014). Lipase maturation factor 1 (Lmf1)
is induced by endoplasmic
reticulum stress through activating transcription factor 6α
(Atf6α) signaling. J. Biol. Chem..

[75] Eichmann T. O., Grumet L., Taschler U., Hartler J., Heier C., Woblistin A., Pajed L., Kollroser M., Rechberger G., Thallinger G. G., Zechner R., Haemmerle G., Zimmermann R., Lass A. (2015). ATGL and CGI-58 are lipid droplet
proteins of the hepatic stellate cell line HSC-T6. J. Lipid Res..

[76] Listenberger L. L., Ostermeyer-Fay A. G., Goldberg E. B., Brown W. J., Brown D. A. (2007). Adipocyte
differentiation-related protein reduces the lipid droplet association
of adipose triglyceride lipase and slows triacylglycerol turnover. J. Lipid Res..

[77] Kim K. H., Oprescu S. N., Snyder M. M., Kim A., Jia Z., Yue F., Kuang S. (2023). PRMT5 mediates FoxO1
methylation and subcellular localization
to regulate lipophagy in myogenic progenitors. Cell Rep..

[78] Luo H., Jiao Q., Shen C., Shao C., Xie J., Chen Y., Feng X., Zhang X. (2023). Unraveling the roles
of endoplasmic reticulum-associated degradation in metabolic disorders. Front. Endocrinol..

[79] Jiao D. L., Chen Y., Liu Y., Ju Y. Y., Long J. D., Du J., Yu C. X., Wang Y. J., Zhao M., Liu J. G. (2017). SYVN1,
an ERAD E3 ubiquitin ligase, is involved in GABAaα1 degradation
associated with methamphetamine-induced conditioned place preference. Front. Mol. Neurosci..

[80] Ho D. V., Chan J. Y. (2015). Induction
of Herpud1 expression by ER stress is regulated
by Nrf1. FEBS Lett..

[81] Choi K., Kim H., Kang H., Lee S. Y., Lee S. J., Back S. H., Lee S. H., Kim M. S., Lee J. E., Park J. Y., Kim J., Kim S., Song J. H., Choi Y., Lee S., Lee H. J., Kim J. H., Cho S. (2014). Regulation of diacylglycerol
acyltransferase 2 protein stability by gp78-associated endoplasmic-reticulum-associated
degradation. FEBS J..

[82] Kim H., Wei J., Song Z., Mottillo E., Samavati L., Zhang R., Li L., Chen X., Jena B. P., Lin J. D., Fang D., Zhang K. (2021). Regulation of hepatic circadian metabolism by the E3 ubiquitin ligase
HRD1-controlled CREBH/PPARα transcriptional program. Mol. Metab..

[83] Gamayun I., O’Keefe S., Pick T., Klein M. C., Nguyen D., McKibbin C., Piacenti M., Williams H. M., Flitsch S. L., Whitehead R. C., Swanton E., Helms V., High S., Zimmermann R., Cavalié A. (2019). Eeyarestatin
Compounds Selectively
Enhance Sec61-Mediated Ca 2+ Leakage from the Endoplasmic Reticulum. Cell Chem. Biol..

