# Supplementary material for: Arctigenin Attenuates Hepatic Stellate Cell Activation via Endoplasmic Reticulum-Associated Degradation (ERAD)-Mediated Restoration of Lipid Homeostasis
Source: J Agric Food Chem. 2025 May 26;73(22):13918–33. doi: 10.1021/acs.jafc.5c01366 (PMC12147166; doi:10.1021/acs.jafc.5c01366)
Supplement: Supplementary file 1 [file jf5c01366_si_001.pdf]

Supporting Information

Arctigenin attenuates hepatic stellate sell activation *via* Endoplasmic Reticulum-associated degradation (ERAD)-mediated restoration of lipid homeostasis

Mengmeng Xia*, Jia Li, Lizbeth Magnolia Martinez Aguilar, Junyu Wang, Maria Camila Trillos Almanza, Yakun Li, Manon Buist-Homan, Han Moshage*

Dept. of Gastroenterology and Hepatology, University Medical Center Groningen, University of Groningen, Groningen, the Netherlands

* Correspondence: a.j.moshage@umcg.nl; m.m.xia@umcg.nl

Supplementary file contains 4 Figures and 1 table.

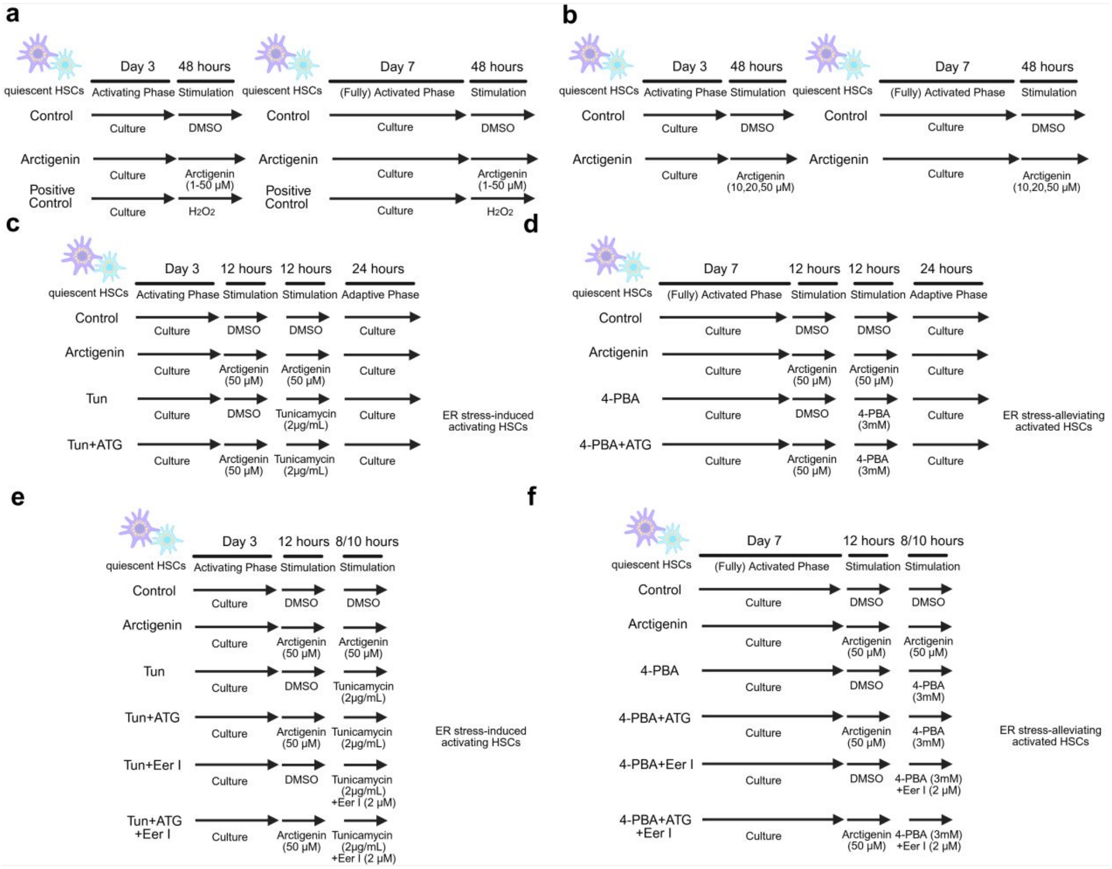

Figure S1. Schematic flowchart illustrating experimental design.

HSC, Hepatic stellate cell; ATG, arctigenin; Tun, tunicamycin; 4-PBA, sodium 4-phenylbutyrate; Eer I, Eeyar-estatin-I; DMSO, Dimethyl sulfoxide.

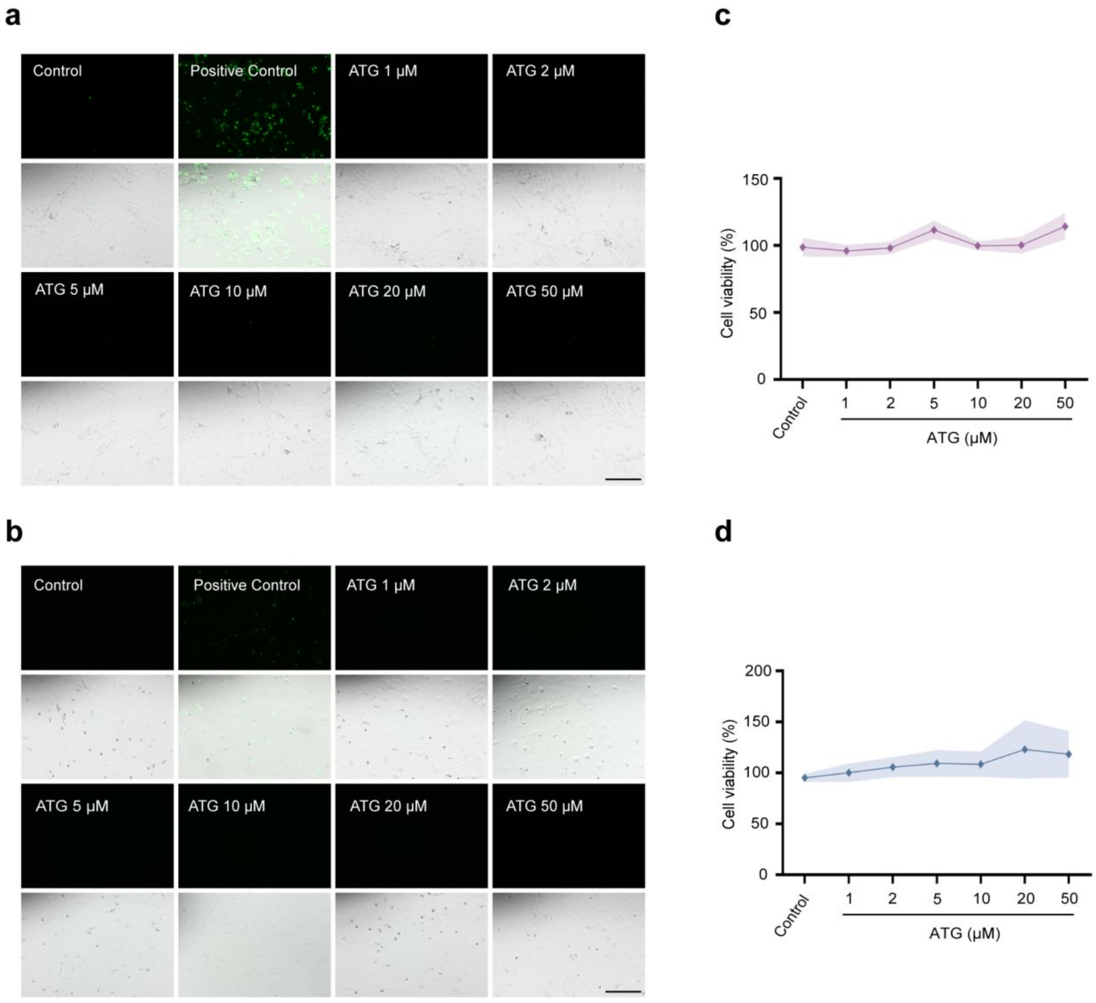

Figure S2. Determination of optimal concentrations of arctigenin.

Activating (Day 3) HSCs and activated (Day 7-10) HSCs (aHSCs) were treated with arctigenin at various concentrations for 48 h. (a) Cytotoxicity of arctigenin on activating HSCs was evaluated using SYTOX Green assay. Green dots represent necrotic cells. Scale bar: 200 μm. (b) Cytotoxicity of arctigenin on activated HSCs was evaluated using SYTOX Green assay. Green dots represent necrotic cells. Scale bar: 200 μm. (c) WST-1 viability assay on activating HSCs treated with different concentrations of arctigenin for 48 h. (d) WST-1 viability assay on activated HSCs treated with different concentrations of arctigenin for 48 h. Control: 1% DMSO; Positive Control: 1 mM hydrogen peroxide (H_2O_2); ATG, arctigenin.

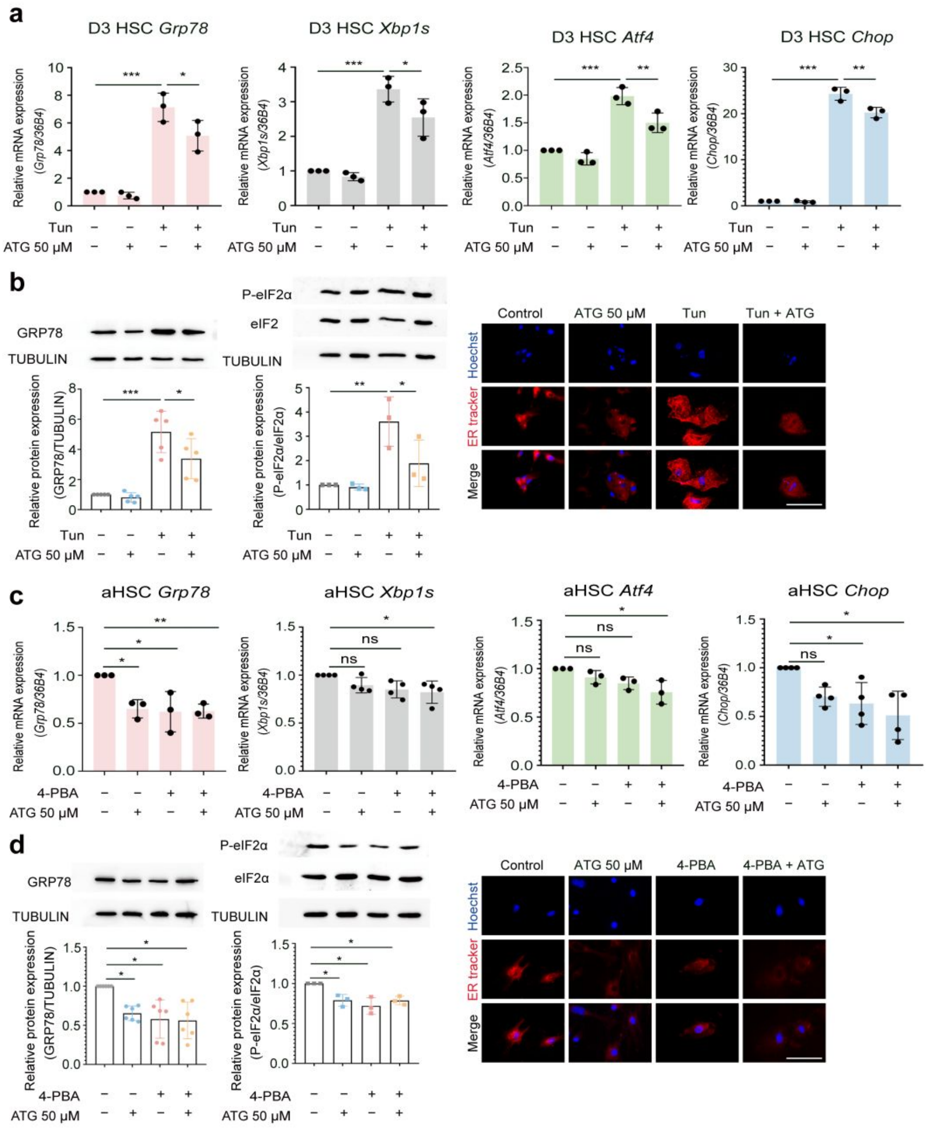

Figure S3. Arctigenin inhibits the UPR in ER stress-exposed hepatic stellate cells.

(a) and (b) Activating (Day 3) HSCs were first treated with arctigenin at 50 μM for 12 h, followed by treatment with 2 μg/mL tunicamycin for 12 h. (a) Real-time PCR analyses of mRNA expression of UPR markers. (b) Western blot analyses of protein expression of UPR markers. Protein intensity was analyzed by Image J software. Live cell ER tracker staining to determine the abundance of ER in activating HSCs. Scale bar: 100 μm. (c) and (d) Activated HSCs were first treated with arctigenin at 50 μM for 12 h, followed by treatment with 3 mM 4-PBA for 12 h. (c) Real-time PCR analyses of mRNA expression of UPR markers. (d) Western blot analysis of protein expression of

UPR markers. Protein intensity was analyzed by Image J software. Live cell ER tracker staining to determine the abundance of ER in aHSCs. Scale bar: 100 μm. HSC, Hepatic stellate cell; D3 HSC, activating HSCs; aHSC, D7 (fully) activated HSCs; ATG, arctigenin; Tun, tunicamycin; 4-PBA, sodium 4-phenylbutyrate. Data are presented as mean ± standard deviation (mean ± SD, n = 3 per group). Statistical significance was determined by one-way ANOVA followed by Tukey's post hoc test. Significance: ns: not significant, $P > 0.05$; *: $P < 0.05$; **: $P < 0.01$.

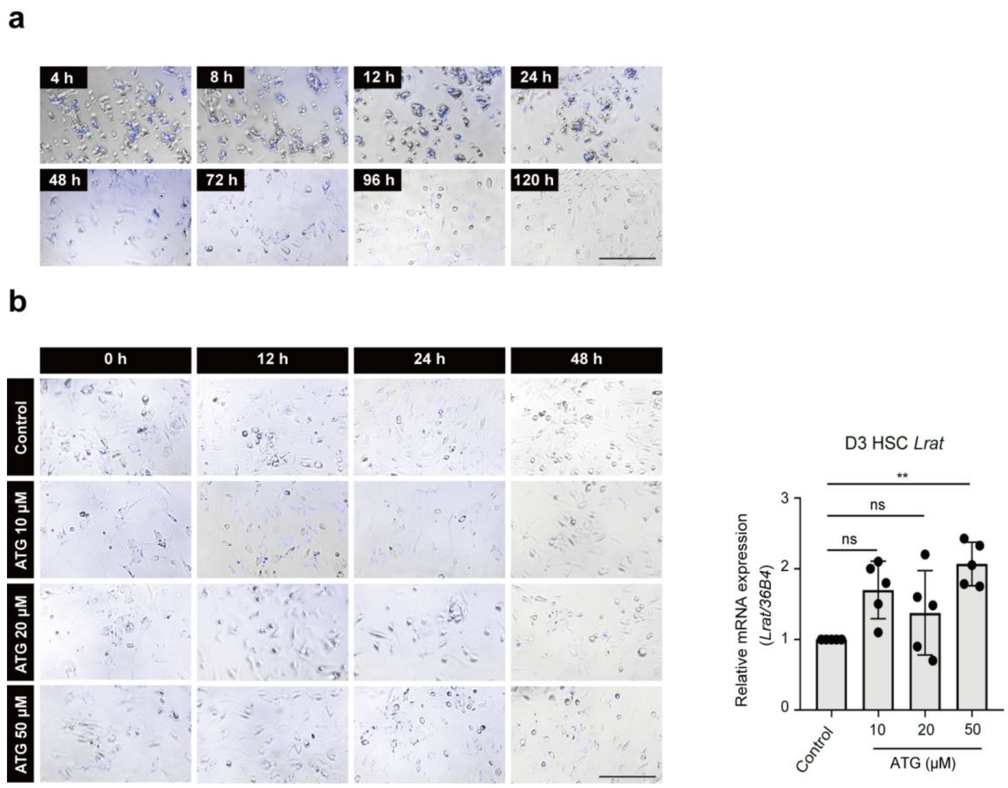

Figure S4. Arctigenin increases retinoid content in activating hepatic stellate cells.

(a) Retinoids inside HSCs were visualized using autofluorescence. Scale bar: 200 μm. (b) Activating HSCs were treated with arctigenin at 10, 20, and 50 μM for different time intervals (left) and for 48 h (right). Retinoids inside HSCs were visualized using auto-fluorescence. Scale bar: 200 μm. Real-time PCR analyses of mRNA expression of HSC quiescence marker Lrat in activating HSCs. HSC, Hepatic stellate cell; D3 HSC, activating HSCs; ATG, arctigenin. Data are presented as mean ± standard deviation (mean ± SD, n > 3 per group). Statistical significance was determined by one-way ANOVA followed by Tukey's post hoc test. Significance: ns: not significant, $P > 0.05$; **: $P < 0.01$.

Table S1. Sequences of primers and probes

Gene	Sequences of primers and probes (5'-3')
36B4	Forward: GCTTCATTGTGGGAGCAGACA<br>Reverse: CATGGTGTTCTTGCCCATCAG<br>Probe: TCCAAGCAGATGCAGCAGATCCGC
P21	Forward: TTGTCGCTGTCTTGCACTCTG<br>Reverse: CGCTTGGAGTGATAGAAATCTGTTA<br>Probe: CTGCCTCCGTTTTCGGCCCTG
P27	Forward: CCTGCGGCAGAAGATTCTTCT<br>Reverse: TGGGCGTCTGCTCCACAG<br>Probe: CCGTCTGAAACATTTTCTTCTGTTCTGTTGGC
Acta2	Forward: GCCAGTCGCCATCAGGAAC<br>Reverse: CACACCAGAGCTGTGCTGTCTT<br>Probe: CTTCACACATAGCTGGAGCAGCTTCTCGA
Col1a1	Forward: TGGTGAACGTGGTGTACAAGGT<br>Reverse: CAGTATCACCCTTGGCACCAT<br>Probe: TCCTGCTGGTCCCCGAGGAAACA
Grp78	Forward: AAAGAAGGTCACCCATGCAGTT<br>Reverse: CAATAGTGCCAGCATCCTTGT<br>Probe: ACTTCAATGATGCACAGCGGCAAGC
Xbp1s	Forward: GCTGAGTCCGCAGCAGGT<br>Reverse: CCCAAAAGGATATCAGACTCAGAATC<br>Probe: CCCAGTTGTCACCTCCCCAGAACATCT
Atf4	Forward: CGGCAAGGAGGATGCCTTT<br>Reverse: ACAGAGCATCGAAGTCAAACTCTTT<br>Probe: CCATTTTCTCCA ACATCCAATCTGTCCC
Chop	Forward: TCCTGTCCTCAGATGAAATTGG<br>Reverse: TCAAGAGTAGTGAAGGTTTTTGATTCT<br>Probe: CACCTA-TATCTCATCCCCAG-GAAACGAAGA
Lrat	Forward: ACTGTGGAACAACTGCGAACAC<br>Reverse: AGGCCTGTGTAGATAATAGACACTAATCC<br>Probe: TTGTGACCTACTGCAGATACGGCTC
Pnpla3	Forward: GTAGCCACTGGATATCTTCATGGA<br>Reverse: TCTTGCTGCCCTGCACTCT<br>Probe: CACCAGCCTGTGGACTGCAGCG
Srebp1c	Forward: GGAGCCATGGATTGCACATT<br>Reverse: CCTGTCTCACCCCCAGCATA<br>Probe: CAGCTCATCAACAACCAAGACAGTGACTTCC
Dgat1	Forward: GGTGCCCTGACAGAGCAGAT<br>Reverse: CAGTAAGGCCACAGCTGCTG<br>Probe: CTGCTGCTACATGTGGTTAACCTGGCCA
Dgat2	Forward: GGGTCCAGAAGAAGTTCCAGAAG<br>Reverse: CCCAGGTGTCAGAGGAGAAGAG<br>Probe: CCCCTGCATCTTCCATGGCCG
Acsl3	Forward: GCCAACGTG-GAAAAGAAAGC<br>Reverse: GTGGACCCACTTGTGTACATGATT<br>Probe: AGCAAACCACTGCCCTCAGATATTGCA

Gene	Sequences of primers and probes (5’-3’)
Elovl5	Forward: TGGCTGTTCTTCCAGATTGGA<br>Reverse: CCCTTTCTTGTTGTAAGTCTGAATGTA<br>Probe: CATGATTTCCCTGATTGCTCTCTTCACAAAC
Ppar-γ	Forward: CACAATGCCATCAGGTTTGG<br>Reverse: GCTGGTCGATATCACTGGAGATC<br>Probe: CCAACAGCTTCTCCTTCTCGGCCTG
Foxo1	Forward: AGATCTACGAGTGGATGGTGAAGAG<br>Reverse: GGACAGATTGTGGCGAATTGAAT<br>Probe: CAGCCCGCCGAG CTGTTGCT

*Continued in Supplementary Table S1
